# Understanding and predicting the geographic distributions of phlebotomine sand flies in and around Europe

**DOI:** 10.1007/s10584-025-04009-z

**Published:** 2025-11-05

**Authors:** Danyang Wang, Anouschka R. Hof, Kevin D. Matson, Frank van Langevelde, CLIMOS data providers, Vít Dvořák, Ognyan Mikov, Ivelina Katerinova, Simona Tchakarova, Maria Antoniou, Jorian Prudhomme, Denis Sereno, Baptiste Vergnes, Nil Rahola, Anne-Laure Bañuls, Maribel Jiménez, Ricardo Molina, Estela Gonzalez, Carla Maia, Ozge Erisoz Kasap, Bulent Alten, Oscar David Kirstein, Shirli Elbaz, Gioia Bongiorno, Luigi Gradoni, Kirami Ölgen, Eduardo Berriatua, Clara Muñoz-Hernandez, Jose Risueño Iranzo, Pedro Pérez Cutillas, Gabriella Gaglio, Emanuele Brianti, Vladimir Ivović, Katja Adam, Sergio Natal, Edwin Kniha

**Affiliations:** Wildlife Ecology and Conservation Group, Department Environmental Sciences, Wageningen University, Wageningen, The Netherlands

**Keywords:** Climate change, Land-use, Moisture, Phlebotomine sand fly, Species distribution modelling, Suitable habitat

## Abstract

Climate and land-use changes influence the transmission of vector-borne diseases by affecting the distribution and survival of disease vectors. Numerous diseases are transmitted by phlebotomine sand flies (Diptera: Psychodidae: Phlebotominae), including leishmaniasis. Several major sand fly-borne diseases are responsible for high global disease burdens and high socio-economic costs. In Europe, over 20 known sand fly vector species are largely confined to the Mediterranean Basin, yet records of sand fly presence further north increase. Global warming is predicted to drive the spread of sand flies to large areas of Europe in the 21th century, an effect likely to be exacerbated by anthropogenic factors. However, the constraints to the geographic distributions of sand flies are not well understood. This study aims to increase the understanding of the drivers of the geographic distributions of sand flies, using species distribution modelling to systematically test links between sand fly occurrences and climatic, land-use, lithological, biodiversity and human population variables in Europe and adjacent Mediterranean regions. We found that moisture is the most important environmental variable both in explaining and in predicting sand fly occurrences. The projected suitable habitats are larger than the current known sand fly distributions, and these habitats are expected to expand due to changes in climate and land-use.

**Supplementary Information:**

The online version contains supplementary material available at 10.1007/s10584-025-04009-z.

## Introduction

Predicting changes in the spatial distribution of organisms due to changes in climate and land-use requires understanding the determinants of spatial distributions. Such predictions and understanding may be especially relevant for disease vectors, as changes in their spatial distributions may have big consequences for human and animal health. Arthropod disease vectors may be particularly sensitive to changes in ambient temperatures, since their life cycle, survival and reproduction are temperature-dependent (Schowalter 2022). One group of arthropod disease vectors that seem to be affected by changes in climate and land-use is the Phlebotomine sand flies (Diptera: Psychodidae: Phlebotominae). Sand flies can transmit numerous parasites, including the protozoan *Leishmania (L.) spp*., which can cause leishmaniasis, and Toscana virus (TOSV), which can cause meningitis and encephalitis (Ayhan and Charrel 2020; Maroli et al. 2013). Leishmaniasis is the second largest parasitic disease in terms of affected human population (Maroli et al. 2013) and the deadliest neglected tropical disease worldwide (Lozano et al. 2012). Of the > 1000 sand fly species that have been described globally, 98 are proven or suspected vectors of *Leishmania spp*. (Maroli et al. 2013), TOSV (Ayhan et al. 2020), or both. In and around Europe, over 20 vector species (Online Resource 1) were largely confined to the Mediterranean countries (Alten et al. 2016; Maroli et al. 2013) until 25 years ago. More recently, small but permanent populations of some sand fly species have been discovered at higher latitudes and altitudes, and these expanded distribution ranges are likely due to climate change (Chalghaf et al. 2018; Maroli et al. 2013; Medlock et al. 2014). With the predicted climate trend, sand flies are expected to reach large parts of north-western and central Europe in the twenty-first century (Koch et al. 2017). Advancing our understanding of the ecology of sand flies, specifically the factors shaping their distributions, will help inform surveillance efforts.

Thus far, studies have modelled sand fly geographic distributions using temperature and precipitation as predictor variables (e.g., Chalghaf et al. 2018; Cunze et al. 2019; Koch et al. 2017), and most have focused on predictive performance. However, anthropogenic factors such as land-use may as well influence sand fly distributions (Medlock et al. 2014; Prudhomme et al. 2024). In addition, the best variables for predicting sand fly distributions do not necessarily ecologically drive sand fly occurrences (Sriboonchitta et al. 2019). Thus, ecological mechanisms that underlie sand fly occurrence are poorly understood.

This study aims to improve our understanding of sand fly ecology and predict the distributions of sand flies in and around Europe. We leverage existing records of species observations and open access datasets to test relations between sand fly occurrences and climatic, land-use, lithological, biodiversity and human population variables on a continental scale. Air temperature and air moisture directly affect sand fly survival and life cycles (Lawyer et al. 2017; Volf and Volfova 2011), and growing season affects vegetation (Brun et al. 2022) that shapes sand fly habitat. Likewise, land-use reflects vegetation type and human disturbance (McKeon et al. 2023), both of which can affect sand fly habitat. Different ecosystems, which can be influenced by lithological variation via matter fluxes (Dürr et al. 2005), provide distinct habitats and resources for sand flies (Ayala 1973; Memmott 1991). Furthermore, a direct link between soil physicochemical properties and sand flies occurrences has been documented in other parts of the world (Kesari et al. 2011; Vivero et al., 2015). Lastly, biodiversity indicators and human population density were used to approximate the sand fly host community composition. Our analyses differentiated the drivers that most affect sand fly occurrences (and thereby help explain *why* sand flies occur where they do) from the variables that best discriminate between locations where sand flies are present or absent (and thereby help predict future sand fly distributions). While the ecological drivers could inform surveillance efforts about potential interventions, predictions could highlight the locations and the timeframe.

## Method

### Sand fly observations

**Fig. 1:**
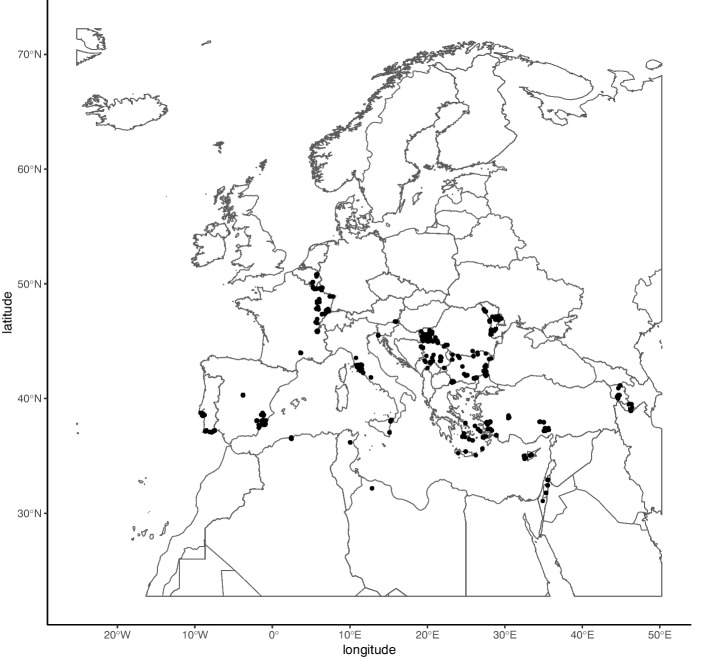
Study area and observation locations

The study area is Europe and neighboring areas (Arnal et al. 2019), bounded by W25°15’ in the west, E50°15’ in the east, N22°45’ in the south, and N72°15’ in the north (Fig. 1). Sand fly observational data from 2005 to 2023 (Online Resource 2) were mainly obtained from previous projects of members of the current team (EU-Horizon project Climate Monitoring and Decision Support Framework for Sand Fly-borne Diseases Detection and Mitigation with COst–benefit and Climate-policy MeasureS; CLIMOS; https://climos-project.eu/). Additional data were collected from published literature (Benabid et al., 2017; Bennai et al., 2018; Cazan et al. 2019; Dokhan et al., 2016; Kavur et al., 2018a; Kavur et al., 2018b; Kuhls et al., 2021; Orshan et al., 2016; Șuleșco et al., 2021; Tsirigotakis et al. 2018; Vaselek et al., 2017, 2019). For a species be included, a minimum threshold of 25 observations (van Proosdij et al. 2016) must be reached after environmental filtering and using only locations that enable spatial cross-validation (both procedures are described in Sect. 2.3). Twelve sand fly species (n = 33 to 284 presence records) were modelled (Table 1).

**Table 1:** The studied sand fly species, their sample sizes, the spatial and the temporal ranges

Species Name	Sample Size	Longitude (decimal degree)	Latitude (decimal degree)	Year
*Phlebotomus (Ph.) papatasi*	284	−7.63—46.4	31.1—47.7	2005–2022
*Ph. perniciosus*	236	−9.28—15.3	36.2—50.8	2010–2023
*Sergentomyia (S.) minuta*	189	−9.28—46.3	32.2—44	2009–2021
*Ph. sergenti*	134	−9.28—46.2	31.8—44.7	2005–2020
*Ph. tobbi*	128	20—35.7	31.8—43.9	2005–2022
*Ph. mascittii*	97	3.63—28.9	36.7—50.8	2010–2023
*Ph. neglectus*	77	13.7—44.6	35—45.8	2011–2023
*Ph. perfiliewi*	77	2.42—35.5	35—47.8	2009–2022
*Ph. ariasi*	75	−9.28—3.69	36.6—44	2011–2018
*S. dentata*	51	24.5—35.7	34.7—41.8	2009–2017
*Ph. simici*	39	19.2—44.5	35.4—44.5	2009–2017
*Ph. alexandri*	33	−2.02—44.7	31.8—43.6	2009–2017

### Environmental variables

Variables related to climate, land-use, lithology, biodiversity and human populations served as potential predictors for the occurrences of sand flies (Online Resource 3). We pre-selected 146 out of a total of 196 climatic variables (at a spatial resolution of 30 arcseconds, means of 1981–2010) (Brun et al. 2022) from Chelsa (https://chelsa-climate.org/) based on their potential ecological relationships with sand flies, numerical nature, and completeness (i.e., few missing values). We used the most recent (i.e., 1981–2010) historical data available to match spatially with sand fly presence records and background data (i.e., 2005–2023) for model training and assessment. Future climatic scenarios, which were available for 80 climatic variables for three periods 2011–2040, 2041–2070 and 2071–2100, were obtained from the general circulation model GFDL-ESM4. This model was found to project realistic climatic conditions for Europe (Palmer et al. 2022). Three Shared Socioeconomic Pathways (SSPs), namely SSP1-2.6 (predicted CO_2_ decline), SSP3-7.0 (predicted CO_2_ increase) and SSP5-8.5 (predicted CO_2_ rapid increase), were used for prediction (IPCC, 2021).

We used 14 land-use variables (at spatial resolution of 0.25 degree and temporal resolution of 1 year) (Chini et al. 2021; Hurtt et al. 2020) from Land-Use Harmonization^2^ (https://luh.umd.edu/data.shtml). Data from 2005 to 2019 was used for model training and testing; future projections for the years 2040, 2070 and 2100 were used for prediction. Here we chose to match land-use covariates with sand fly presence records and background data in space and in time (i.e., year) instead of averaging across years (i.e., resembling climatic data) to preserve variations in predictor variables and to test their relationships with sand fly occurrences. In addition, a global lithological map (at spatial resolution of 30 arcminutes) (Hartmann and Moosdorf 2012) provided data on the type and resistance to weathering and erosion of the rock in an area (Dürr et al. 2005).

We tested the impact of 34 biodiversity indicators (at spatial resolution of 1 degree) (Baisero and Rondinini 2023; Hill and Purvis 2023; Martins et al. 2018; Martins and Pereira 2022) obtained from The Group on Earth Observations Biodiversity Observation Network (GEO BON, https://geobon.org/). We also included human population density (at spatial resolution of 30 arcseconds and temporal resolution of 5 years interval between 2000 and 2020) from NASA's Socioeconomic Data and Applications Center (SEDAC, https://earthdata.nasa.gov/centers/sedac-daac) (Center for International Earth Science Information Network - CIESIN - Columbia University 2018). The layers of future land-use and lithology (kept constant from present to future) were rescaled to 30 arcseconds to match up the spatial resolution of other variables for prediction.

The spatial and temporal resolutions of the environmental variables were the highest that were available to us. Although they were not sufficient to proxy microhabitat which could be important in the ecological processes at finer scales. The temporal extents of the environmental variables partly overlapped with the time span of the observations and were the best match we could obtain.

### Modelling

**Fig. 2:**
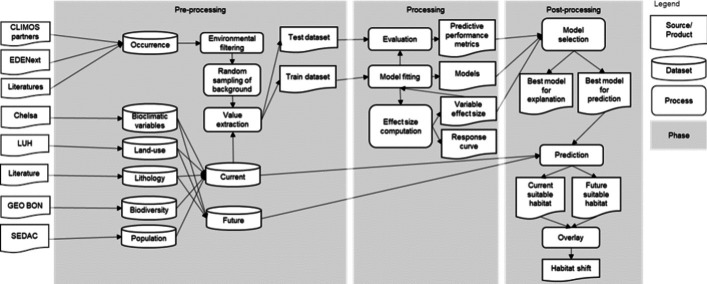
Modelling process (adapted from Wang et al. (2022))

The modelling procedure consisted of three steps (Fig. 2). In the pre-processing stage, sand fly count data were converted to presence data. This presence data is spatially and temporally biased as induced by research-driven and project-specific surveys. For example, 58.8% of observations were made where mean annual air temperature (BIO1) was 18.8 °C and annual precipitation (BIO12) was 511.7 mm. To correct for this sampling bias, we applied environmental thinning (Varela et al. 2014) on sand fly presence records per species. For each unique environmental condition, considering all covariates, one record was randomly retained using the sample() function in R ‘base’ (R Core Team 2024). This way, all environmental conditions in which species occur were weighted equally regardless how many observations has been made (due to sampling bias). Even if a habitat generalist is more often observed in cities than in the nature due to e.g., accessibility, the entire niche of the species is well distinguished from background condition. Afterwards, 100,000 background points (Renner et al. 2015) were randomly sampled within the study area using the st_sample() function of the ‘sf’ package (Pebesma 2018), and were randomly assigned a year number between 2005–2023 (i.e., the temporal range of presence data). An equal number of background data points were assigned to each year. Afterwards, the values of historical environmental variables were extracted at the locations and in the periods of sand fly observations using the function extract() in the ‘terra’ package (Hijmans 2023) and were standardized. The variables for future predictions were also standardized using the means and the standard deviations of the historical dataset.

In the processing phase, models were fitted using maximum entropy (Maxent) (Phillips et al. 2006) in RStudio (R Core Team 2024) and the ‘dismo’ package (Hijmans et al. 2022). We used all feature classes except threshold (Phillips et al. 2017). We first determined the value of the regularization multiplier per species before model fitting (Online Resource 4, eight species with regularization multiplier nine, three species seven, and one species five). After feature class and regularization multipliers were determined, we fitted single variable models for all variables (196 models per studied species), computed variable effect size using the lambda file of Maxent (Online Resource 5), and spatially cross-validated the models (Online Resource 4). Spatial cross-validation was chosen to mitigate influence from spatial autocorrelation (Bahn and McGill 2013; Hijmans 2012), which can be caused by, among others, the limited flight capacity of sand flies (Maroli et al. 2013). Two model evaluation statistics were computed using the evaluate() function in the ‘dismo’ package (Hijmans et al. 2022): the area under the receiver operating characteristic curve (AUC) and True Statistic Skills (TSS) (Allouche et al. 2006; Fielding and Bell 1997).

We also fitted and cross-validated models using the spatial blocks for all possible two-variable combinations of the variables that were either static or with future projections for the chosen horizons and SSPs (i.e., 95 variables). These variables included land-use, lithological and a part of the climatic variables. Biodiversity indices had either no future scenarios or had projections in 2050 with different SSPs and could not be used for predictions of the selected horizons (i.e., 2040, 2070 and 2100) and scenarios (i.e., SSP1-2.6, SSP3-7.0, SSP5-8.5). Human population density did not have a future projection. To avoid multicollinearity, the two-variable combinations must have a variance inflation factor (VIF) ≤ 10 (Akinwande et al. 2015) as computed in the ‘usdm’ (Naimi et al. 2014). The chosen VIF threshold was higher than the generally accepted VIF ≤ 5 for regression models (Akinwande et al. 2015), since Maxent can deal relatively well with correlated variables (Elith et al. 2011). We limited the number of variables in a model to two to avoid high computational expenses.

In the post-processing phase, we distinguished explanatory ecological drivers from predictive variables by using different selection criteria. The best explaining variables were taken as those with the highest effect sizes and with AUC > 0.7; the best predictive models were taken as those with the highest AUC and TSS. The best predictive models were used to project suitable habitats under current conditions and future (i.e., under SSP1-2.6, SSP3-7.0, SSP5-8.5) conditions for the three future time horizons (i.e., 2040, 2070, 2100). These habitat suitability maps were converted to binary maps using threshold values that maximize the sum of sensitivity and specificity (Liu et al. 2013). We then predicted habitat shifts by comparing current suitable habitats with future habitats. Areas where it is currently unsuitable for sand flies to occur but will become suitable habitat in the future expect range expansion; areas where current suitable habitats will become unsuitable for sand flies to inhabit expect range contraction. In addition, we overlayed the current and the future habitat projections of the five proven vectors *L. infantum* to illustrate expected changes in vector diversity.

## Results

When the 196 covariates were considered in isolation, the variables with the largest effect sizes were mostly related to moisture (9 of 12 species), and to a lesser extent to temperature (3 of 12 species; Table 2). In particular, climate moisture indices had the largest effect sizes for seven species. All but one of the nine species responded unimodally to these moisture related drivers; *Phlebotomus (Ph.) tobbi* responded positively to climate moisture index range. Temperature-related variables had the largest effect sizes for three species, all of which responded unimodally.

**Table 2:** Single variable models with the largest effect sizes and the largest discrimination power, and two-variable models with the best predictive performance, their predictive metrics and thresholds to convert suitability maps to binary maps

Species Name	Single Variable with the Largest Effect Size	Effect Size	Response curve	Single Variable Model with the Best Predictive Performance	AUC	TSS	Parsimonious Model with the Best Predictive Performance	AUC	TSS	Threshold
*Ph. alexandri*	Monthly climate moisture index in September	54	unimodal	Mean monthly climate moisture index	0.85	0.71	Snow cover days Mean maximum air temperature of March	0.87	0.74	0.64
*Ph. ariasi*	Monthly climate moisture index in June	30	unimodal	Mean climate moisture index in July	0.91	0.85	C3 annual crops C3 perennial crops	0.93	0.87	0.34
*Ph. mascittii*	Mean annual air temperature	20	unimodal	Net primary productivity	0.82	0.66	Net primary productivity Snow cover days	0.90	0.81	0.23
*Ph. neglectus*	Mean climate moisture index in August	31	unimodal	Mean air temperature in May	0.89	0.76	Mean daily mean air temperatures of the warmest quarter Annual precipitation amount	0.91	0.82	0.50
*Ph. papatasi*	Mean potential evapotranspiration in April	14	unimodal	Mean near-surface relative humidity in February	0.90	0.79	Accumulated precipiation amount on growing season days Mean maximum air temperature of May	0.91	0.81	0.47
*Ph. perfiliewi*	Mean climate moisture index in June	34	unimodal	Mean vapor pressure deficit in March	0.93	0.84	Accumulated precipiation amount on growing season days Mean air temperature of May	0.95	0.88	0.51
*Ph. perniciosus*	Mean air temperature in December	17	unimodal	Mean maximum air temperature in October	0.83	0.70	Number of growing degree days > 0 °C Urban land	0.89	0.76	0.60
*Ph. sergenti*	Minimum monthly vapor pressure deficit	34	unimodal	Mean potential evapotranspiration in March	0.91	0.81	Secondary mean biomass carbon density Mean air temperature of August	0.92	0.83	0.47
*Ph. simici*	Mean climate moisture index in September	40	unimodal	Mean vapor pressure deficit in October	0.91	0.81	Total precipitation of March Mean maximum air temperature of June	0.94	0.88	0.42
*Ph. tobbi*	Annual range of monthly climate moisture index	32	positive	Species richness of non-forest birds in 2015	0.93	0.81	Accumulated precipiation amount on growing season days Total precipitation of August	0.94	0.88	0.24
*S. dentata*	Mean air temperature in October	37	unimodal	Mean potential evapotranspiration in September	0.96	0.91	Net primary productivity Total precipitation of September	0.95	0.92	0.56
*S. minuta*	Mean climate moisture index in March	27	unimodal	Total precipitation in July	0.92	0.78	Total precipitation of October Mean maximum air temperature of February	0.94	0.84	0.20

When considering the discrimination power of single variables, moisture-related covariates (i.e., climate moisture index, relative humidity, vapor pressure deficit, potential evapotranspiration and precipitation) best predicted the suitable habitat for eight of the 12 species. In these cases, AUC ranged from 0.85 to 0.96, and TSS ranged from 0.71 to 0.91. Temperature-related variables (i.e., mean and max air temperature) best predicted the suitable habitat for two of the 12 species, with AUC ranging between 0.83 and 0.89 and TSS between 0.70 and 0.76. Net primary productivity best predicted the suitable habitat of *Ph. mascittii* (AUC = 0.82; TSS = 0.66); a biodiversity index (i.e., species richness of non-forest birds) best predicted the suitable habitat of *Ph. tobbi* (AUC = 0.93; TSS = 0.81).

The best two-variable predictive models (selected from 95 variables) contained either climatic, or land-use, or a combination of climatic and land-use variables (Table 2). A combination of different climatic variables (i.e., moisture-related, temperature-related and net primary productivity) can best predict the suitable habitat for seven of the 12 studied species (AUC ranged between 0.90 and 0.95, TSS between 0.81 and 0.92). Temperature-related variables alone can best predict the suitable habitat for *Ph. alexandri* (AUC = 0.87, TSS = 0.74); moisture-related variables alone for *Ph. tobbi* (AUC = 0.94, TSS = 0.88). Climatic and land-use variables combined can best predict the suitable habitat for two species (AUC ranged from 0.89 to 0.92, TSS from 0.76 to 0.83). Land-use variables alone can best predict the suitable habitat for *Ph. ariasi* (AUC = 0.93, TSS = 0.87).

**Fig. 3:**
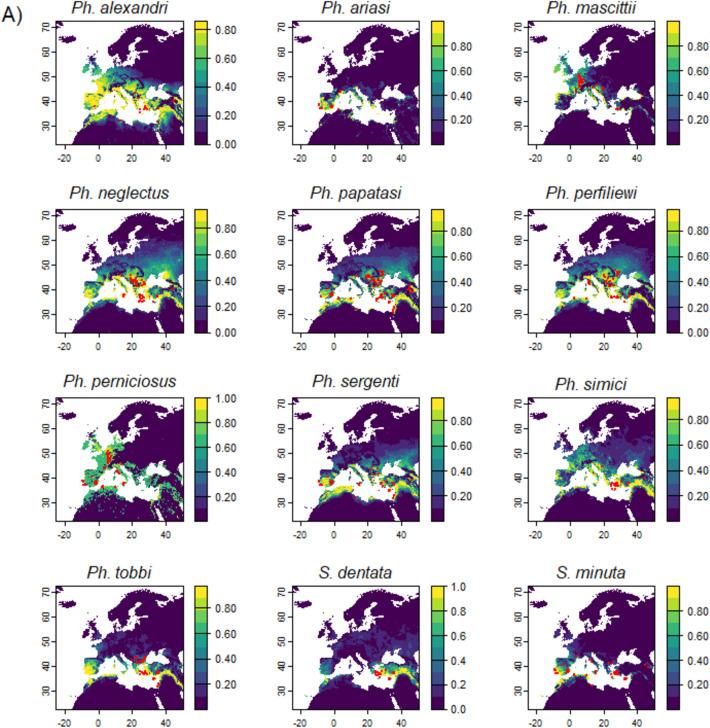

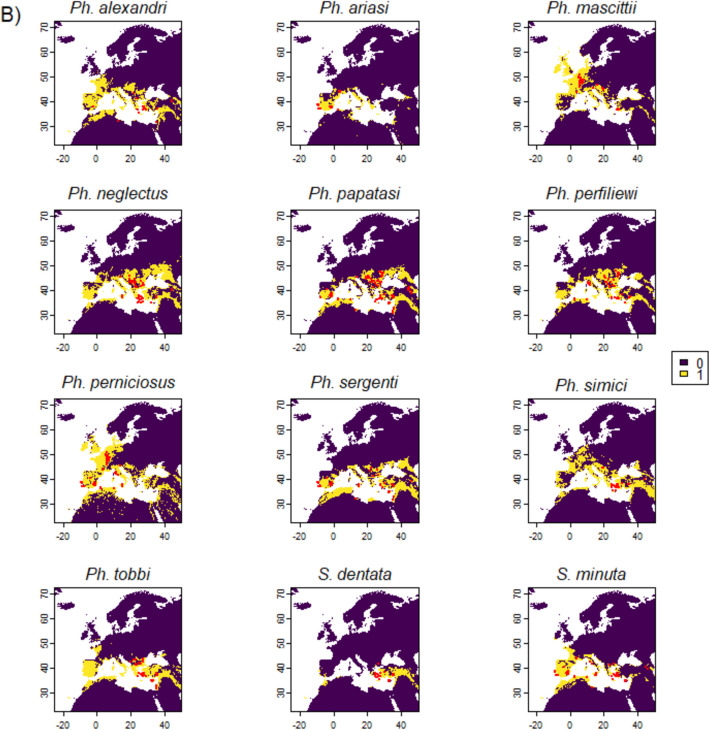
Sand fly habitat suitability (A) and binary suitable habitat maps (B) under current environmental conditions. Colours show the probability of occurrence. Dots are presence observations.

The parsimonious predictive models projected larger suitable habitats than the observed distribution ranges for the studied species under current environmental conditions (Fig. 3).

**Fig. 4:**
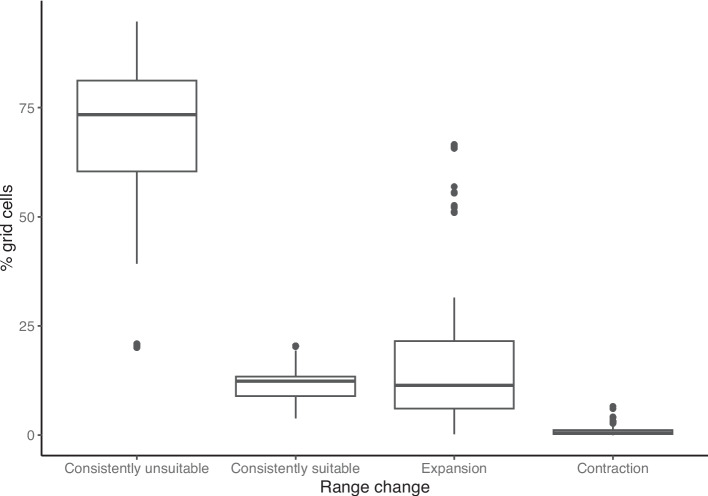
Percentage of grid cells in the study area that is predicted with range changes across species, horizons (i.e., 2040, 2070, 2100) and scenarios (i.e., SSP1-2.6, SSP3-7.0, SSP5-8.5).

Sand flies’ suitable habitats are expected to expand in the future compared to now (Fig. 4). Across the 12 species, the three future horizons and three scenarios, an average of 68.6% of the area in the study area will remain unsuitable and an average of 11.6% will stay suitable for sand flies to occur. About 19% of the study area is currently unsuitable habitat for sand flies but will become suitable for one or more sand flies to inhabit (i.e., range expansion), whereas the areas where suitable habitat will become unsuitable for sand flies (i.e., range contraction) are negligible.

**Fig. 5:**
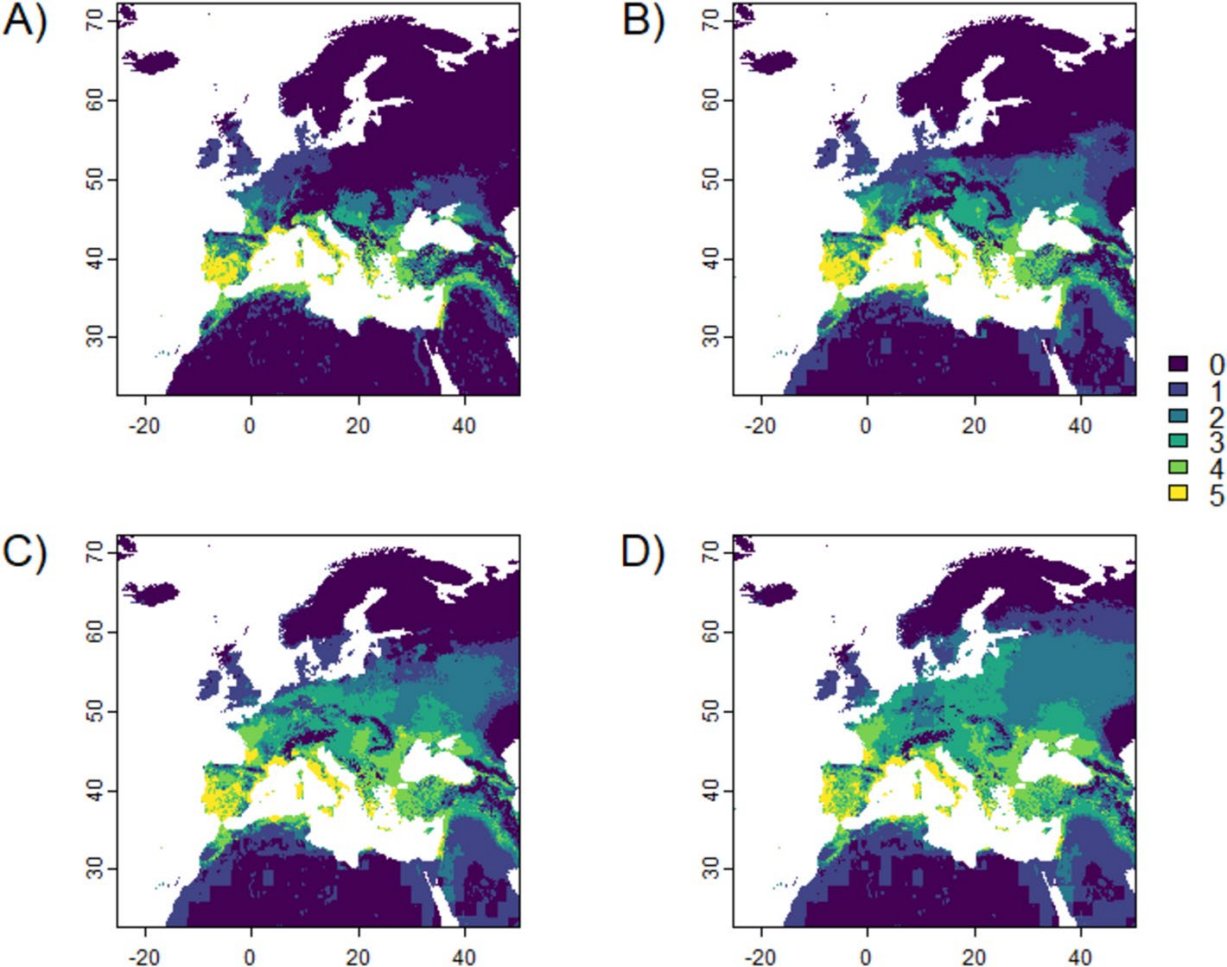
Suitable habitats, now (A) and in the future under SSP3-7.0 (2040 (B), 2070 (C), 2100 (D)), associated with vector species richness for those sand flies that can transmit *L. infantum* (i.e., *Ph. ariasi, Ph. neglectus, Ph. perniciosus, Ph. perfiliewi, Ph. tobbi* (Online Resource 1)).

Overlaying the binary suitability maps of the five confirmed vector species of *L. infantum*, the major pathogen causing visceral leishmaniasis in Europe, resulted in a map of *L. infantum* vector species richness (Fig. 5). Larger ranges are predicted to host more vector species under future climatic and land-use conditions compared to now. Regions that now already have a high vector richness (including southwest Iberia, the south and southwest coasts of France, coastal regions in Italy and in the Balkans, and west and central Turkey, Fig. 5A) are expected to see *L. infantum* vector species hotspots expanding.

## Discussion

Phlebotomine sand flies are vectors of leishmaniasis and several viral neuro-invasive diseases that are responsible for heavy disease burdens and socio-economic impact (Lenk et al. 2018; Lozano et al. 2012; Maroli et al. 2013). The emergence and re-emergence of sand fly-borne diseases in Europe (Maia 2024) and the expectation of range expansion of sand flies in Europe necessitate a better understanding of the ecology of these organisms. We explored 196 environmental variables using machine learning techniques and identified variables that are ecologically the most relevant for each studied sand fly species. We also constructed parsimonious predictive models for all studied species and generated potential distribution predictions under current and future environmental conditions.

Occurrences of the majority of the studied sand fly species (9 of 12) were best explained by moisture-related climatic variables, with the others being explained by temperature-related variables. This result suggests that, although temperature greatly controls the development speed of sand flies (Killick-Kendrick 1999; Maroli et al. 2013), moisture most affects where they occur on large temporal-spatial scale (i.e., (multi-)annual, reginal and beyond). Among the moisture-related variables, the most identified was climate moisture index (7 species), a climatic measure combining precipitation and potential evapotranspiration (Brun et al. 2022) and informing about net water availability to organisms like sand flies. In contrast to relative humidity or precipitation, which may influence sand fly activity in a more transient and local manner as shown in earlier field studies e.g., (Cazan et al. 2019; Gálvez et al. 2010; Kniha et al. 2021; Muñoz et al. 2018; Prudhomme et al. 2015; Tsirigotakis et al. 2018), climatic moisture index had the largest impact on sand fly presence for the studied scale. Biodiversity indices (i.e., (weighted) relative changes in bird species richness) had large effect sizes for a few sand fly species (i.e., *Ph. ariasi, Ph. mascittii, Ph. perniciosus, Sergentomyia (S.) minuta*), underscoring an important role of wildlife being hosts for sand flies (Veiga et al. 2024). In addition, bird richness could be a competent bioindicator for environmental conditions (Mekonen 2017) for sand flies. However, biodiversity indices could not reasonably predict sand fly occurrences (i.e., AUC < 0.7), possibly influenced by the coarse resolution of these variables (i.e., 1 degree).

The most effective environmental variables, i.e., moisture-related climatic variables, representing one dimension of the ecological niches of the studied species, do not mirror the taxonomic relationships of sand flies but are more related to their spatial patterns. For example, *Ph. alexandri* and *Ph. simici* belong to different subgenera but both are most affected by the climate moisture index in September and both prefer where this index is slightly lower than its mean value (Online Resource 6). These two sand fly species are caught in different sites (with *Ph.alexandri* being more widely distributed than *Ph. simici*), but their modelled suitable habitats largely overlap (Fig. 3). The much more restricted realized distribution of *Ph. simici* could thus be driven by variables not included in the current study. In addition, *Ph. ariasi* and *Ph. perniciosus* are two sister species under subgenus *Larroussius.* Both are found in Iberia, France, Italy and northwest Africa (European Centre for Disease Prevention and Control n.d.). These two species are most affected by two highly negatively correlated variables (i.e., climatic moisture index in June and mean air temperature in December respectively, correlation coefficient = −0.86) with similar response curves (only that the niche of *Ph. perniciosus* is slightly wider and more available in the landscape, Online Resource 6). The complimentary niches of *Ph. ariasi* and *Ph. perniciosus* may explain their sympatric distributions.

Similar to the most effective variables in explaining sand fly occurrences, variables that could best distinguish presence sites from background locations were mostly related to air moisture and water availability (8 of 12 species). These measurements of moisture (i.e., climate moisture index, potential evapotranspiration, vapor pressure deficit, humidity, precipitation) resulted in high discrimination power, possibly because they drive and thus well approximate ecosystem properties (Novick et al. 2024; Siepielski et al. 2017). We found that the variables that were the most ecologically relevant for sand fly presence (i.e., variables with the largest effect sizes) are often not the same as the ones with the largest discrimination power (i.e., variables with the highest AUC and TSS). Variables with large effect sizes may fit the data less well (e.g., with larger error) and thus can less well discriminate sites where sand flies are present from background locations. In general, we show that species distribution models can be used for different purposes such as exploration, inference and prediction (Tredennick et al. 2021). It is advisable to select models depending on the research questions (Tredennick et al. 2021), which in our case related to selecting for effect size for ecological explanation and selecting for predictive performance for prediction purpose.

Although the exact measures for the largest effects and for the highest discrimination power are different, we found that moisture is the most important environmental factor for sand fly occurrences. The lack of future scenarios for many of the moisture-related variables (only precipitation has future projections) hampers projection of potential habitat into the future. For prediction purposes, we had to select models from variables with future projections and the outcomes are mainly a combination of climatic and land-use variables.

The projected suitable habitats under current environmental conditions are generally beyond the observed distributions of sand flies (European Centre for Disease Prevention and Control n.d.). This apparent paradox could result from the current realized distributions of sand flies being largely formed via evolutionary history and paleoclimatic events (Cruaud et al. 2021; Esseghir et al. 2000). Occupation of the full potential range may be impeded by (biotic) factors that are not a part of our model, including microhabitat, niche width, dispersal, habitat availability and stability, and interspecific competition (Godsoe and Harmon 2012; Pulliam 2000). Long distance dispersal is rare in sand flies (Orshan et al. 2016; Pérez-Cutillas et al. 2020), and passive transportation is considered unlikely due to their fragility and sensitivity to desiccation (European Centre for Disease Prevention and Control 2020). Furthermore, previously reported “range expansion” must take into consideration increases in sampling efforts in non-endemic regions (e.g., Risueño et al. 2024). The observed non-overlapping distributions on large geospatial scale are not likely a result of host preference, since many largely allopatric (and partly sympatric) species (e.g., *Ph. ariasi*, *Ph. perniciosus*, *Ph. perfiliewi* and *Ph. neglectus*) are opportunistic feeders with a large host range (Bongiorno et al. 2003; Guy et al. 1984; Veiga et al. 2024; Velo et al. 2005). Furthermore, anthropophilic behaviour (Chaskopoulou et al. 2016; Veiga et al. 2024) should facilitate sympatric distribution thanks to host availability. Other types of interspecific competition are largely unknow for *Phlebotomus spp.* However, experiments on other *Diptera spp.* show that larvae competition can affect adult emergence (e.g., Schneider et al. 2000; Wasti et al. 1975; Werenkraut et al. 2008) and can therefore determine their distributions (Rodrıguez-Castañeda et al. 2017). Future studies on competition among sand fly species can help advance our knowledge on their ecology and distributions.

We projected larger suitable sand fly habitat compared to an earlier modelling exercise. Koch et al. (2017) used six predetermined climatic variables and applied ensemble prediction using a maximum of 10 models (Koch et al. 2017). Their projections of current sand fly habitat were essentially restricted to the observed ranges (Koch et al. 2017). An alternative approach to project species realized distributions (instead of suitable habitat) could be using absence observations instead of pseudo-absence points for species distribution modelling (Brown and Griscom 2022). Our current projections for *Ph. alexandri*, *Ph. perniciosus, Ph. sergenti* extent to higher latitudes compared to the projections of Koch et al. (2017). This difference likely reflects the changing climate. To recap, we used more recent observations (2005–2023) and explanatory variable data (1981–2010) compared to Koch et al. (2017) (observations from 1984; bioclimatic variables from 1960–1990 (Koch et al. 2017)).

Our models predicted future habitat expansion to higher latitudes under the most scenarios for the majority of the studied species, underscoring previous predictions of northwards shifting of suitable climate for sand flies in Europe (Koch et al. 2017). Poleward, eastward and to a lesser extent southward range expansion is also predicted for the richness of vector species of *L. infantum*, the major pathogen causing visceral leishmaniasis in Europe. Despite these predictions, the observed sand fly distributions lag behind the shifting suitable habitat. A recent field survey suggests that the northern border of *Phlebotomus spp.* distribution in central-west Europe is in Luxemburg (Risueño et al. 2024). In addition, some longitudinal surveys provided evidence for local adaptation in the form of prolonged or multimodal active season (unpublished data). It is therefore uncertain if, and if so in which time frame, sand flies will track their suitable habitats and migrate to novel regions. As comparison, tick (*Ixodes ricinus*) has been both predicted (Alkishe et al. 2017) and observed (Jaenson et al. 2012) expanding its geographic distribution in Europe, probably partially due to passive transport on its hosts which is not the case of rapidly feeding sand flies.

Overall, our results show that moisture is the most important factor for sand fly occurrences. Areas larger than the current known distributions are suitable for sand fly species in terms of climate and land-use. Furthermore, these areas are expected to expand due to changes in climate and land-use. It is, however, uncertain to what extent and at what rate sand flies will track their suitable habitat northwards to spread to large regions in Europe. Experiments on interspecific competition among sand flies are needed to advance our understanding on sand fly ecology and distributions. In addition, surveillance in non-endemic regions (both presence and absence observations) will provide ground truth for realized distribution ranges, especially for vector species with few observations (e.g., *Ph. balcanicus*, *Ph. longicuspis*). Unlike invasive mosquitos (e.g., *Aedes spp.*) that are invading temperate regions and transmitting zoonoses (e.g., yellow fever, dengue, West Nile Virus) (Giunti et al. 2023), the geographic distribution of phlebotomine sand flies may not solely drive the spread of sand fly-borne diseases. Rather, the activity patterns of sand flies and host-vector-pathogen interplay could be modified by climate and land-use changes (Rizzoli et al. 2019). Future studies on vector abundance, seasonality and vector competence could contribute to disease risk assessment. Additionally, exposure risks are expected to increase due to anthropogenic global changes (Cosma et al. 2024). Finally, vulnerability for sand fly-borne diseases is likely heightened by demographic structure change and other immunosuppressive factors (Maia 2024). All these factors contribute to the spread of sand fly-borne diseases. Future research focused on these topics will help contribute to sand fly-borne diseases preparedness.

## Supplementary Information

Below is the link to the electronic supplementary material.

Supplementary file1 (PDF 73 KB)

Supplementary file2 (PDF 136 KB)

Supplementary file3 (XLSX 15 KB)

Supplementary file4 (PDF 91 KB)

Supplementary file5 (PDF 124 KB)

Supplementary file6 (ZIP 115 KB)

## Data Availability

Sand fly observational data sources are disclosed in Online Resource 2. Environmental data sources are disclosed in the text.
