# Supplementary material for: Understanding and predicting the geographic distributions of phlebotomine sand flies in and around Europe
Source: Clim Change. 2025 Nov 5;178(11):205. doi: 10.1007/s10584-025-04009-z (PMC12589297; doi:10.1007/s10584-025-04009-z)
Supplement: Supplementary file 1 — Supplementary file1 (PDF 73 KB) [file 10584_2025_4009_MOESM1_ESM.pdf]

Supplementary Information

Table 1. Major SFBDs pathogens in and around Europe, their proven and suspected vectors and reservoirs.

Pathogen	Distribution	Vector*	Reservoir*	Reference
L. infantum	Mediterranean Basin	*Ph. ariasi,* *Ph. balcanicus,* *Ph. halepensis,* *Ph. kandelakii,* *Ph. langeroni,* *Ph. longicuspis,* *Ph. longiductus,* *Ph. mascittii,* *Ph. neglectus,* *Ph. perfiliewi,* *Ph. perniciosus,* *Ph. syriacus,* *Ph. transcaucasicus,* *Ph. tobbi*	*Dog (Canis lupus familiaris), cat (Felis catus), red fox (Vulpes vulpes), golden jackal (Canis aureus), wolf (Canis lupus), badger (Meles meles), common genet (Genetta genetta), pine marten (Martes martes), wildcat (Felis silvestris), Iberian lynx (Lynx pardinus), Cranada hare (Lepus granatensis), European rabbit (Oryctolagus cuniculus), black rat (Rattus rattus), brown rat (Rattus norvegicus)*	[1–4]
L. major	North Africa and the Middle East	*Ph. bergeroti,* *Ph. papatasi*	*Fat sand rat (Psammomys obesus), gerbils (Meriones spp., M. shawi, Gerbillus spp.), Algerian hedgehog (Ateleris algirus), desert hedgehog (Paraechinus aethiopicus), cat (Felis catus), Social vole (Microtus socialis), Short-tailed bandicoot rat (Nesokia indica)*	[2,4]
L. tropica	North Africa and the Middle East	*Ph. arabicus,* *Ph. chabaudi,* *Ph. sergenti*	*Rock hyrax (Procavia capensis), North African gundi (Ctenodactylus gundi), cat (Felis catus), Human (Homo sapiens)*	[2,4]
L.donovani	Cyprus and Turkey	*Ph. alexandri,* *Ph. galilaeus,* *Ph. tobbi*	*Human (Homo sapiens)*	[2,4,5]
Toscana virus (TOSV)	Mediterranean Basin and North Africa	*Ph. longicuspis,* *Ph. neglectus,* *Ph. papatasi,*	*Unknown*	[4,6]

	Ph. perfiliewi,
	Ph. perniciosus,
	Ph. sergenti,
	Ph. tobbi,
	S. minuta

* proven or suspected

Reference

1. Alten, B. *et al.* Seasonal Dynamics of Phlebotomine Sand Fly Species Proven Vectors of Mediterranean Leishmaniasis Caused by Leishmania infantum. *PLoS Negl Trop Dis* **10**, (2016).
2. Maroli, M., Feliciangeli, M. D., Bichaud, L., Charrel, R. N. & Gradoni, L. Phlebotomine sandflies and the spreading of leishmaniases and other diseases of public health concern. *Med Vet Entomol* **27**, 123–147 (2013).
3. Obwaller, A. G. *et al.* Could Phlebotomus mascittii play a role as a natural vector for Leishmania infantum? New data. *Parasit Vectors* **9**, 1–6 (2016).
4. Moriconi, M. *et al.* Phlebotomine sand fly–borne pathogens in the Mediterranean Basin: Human leishmaniasis and phlebovirus infections. *PLoS Neglected Tropical Diseases* vol. 11 Preprint at https://doi.org/10.1371/journal.pntd.0005660 (2017).
5. Özbilgin, A. *et al.* Leishmaniasis in Turkey: Visceral and cutaneous leishmaniasis caused by Leishmania donovani in Turkey. *Acta Trop* **173**, 90–96 (2017).
6. Ayhan, N., Prudhomme, J., Laroche, L., Bañuls, A. L. & Charrel, R. N. Broader Geographical Distribution of Toscana Virus in the Mediterranean Region Suggests the Existence of Larger Varieties of Sand Fly Vectors. *Microorganisms* **8**, (2020).
