# Supplementary material for: Understanding and predicting the geographic distributions of phlebotomine sand flies in and around Europe
Source: Clim Change. 2025 Nov 5;178(11):205. doi: 10.1007/s10584-025-04009-z (PMC12589297; doi:10.1007/s10584-025-04009-z)
Supplement: Supplementary file 2 — Supplementary file2 (PDF 136 KB) [file 10584_2025_4009_MOESM2_ESM.pdf]

Supplementary Information 2. Sand fly data sources.

Dataset	Source
Austria dataset	Kniha et al., 2020, 2021
Bulgaria dataset	Dvorak et al., 2020
EDENext* Crete and Cyprus dataset	Dvořák et al., 2020 and unpublished data
EDENext* France dataset	Alten et al., 2016; Prudhomme, 2015; Prudhomme et al., 2015
EDENext* Madrid dataset	González et al., 2017
EDENext* Portugal dataset	Alten et al., 2016
EDENext* Turkey dataset	Alten et al., 2016
Israel dataset	Unpublished data
Italy dataset	Gradoni et al., 2022; Morosetti et al., 2020
Mugla dataset	Pekağırbaş et al., 2021
Murcia dataset	MuÑoz et al., 2018; Muñoz et al., 2019, 2021
Sicily dataset	Abbate et al., 2020
Slovenia dataset	Unpublished data

*This study used data produced during the EU-funded EDEN and EDENext projects.

Additional sand fly observational data were collected from published literature (Benabid et al., 2017; Bennai et al., 2018; Cazan et al., 2019; Dokhan et al., 2016; Kavur, Arikan, et al., 2018; Kavur, Artun, et al., 2018; Kuhls et al., 2021; Orshan et al., 2016; Șuleșco et al., 2021; Tsirigotakis et al., 2018; Vaselek et al., 2017, 2019).

References

Abbate, J. M., Maia, C., Pereira, A., Arfuso, F., Gaglio, G., Rizzo, M., Caracappa, G., Marino, G., Pollmeier, M., Giannetto, S., & Brianti, E. (2020). Identification of trypanosomatids and blood feeding preferences of phlebotomine sand fly species common in Sicily, Southern Italy. *PLOS ONE*, *15*(3), e0229536. https://doi.org/10.1371/JOURNAL.PONE.0229536

Alten, B., Maia, C., Afonso, M. O., Campino, L., Jiménez, M., González, E., Molina, R., Bañuls, A. L., Prudhomme, J., Vergnes, B., Toty, C., Cassan, C., Rahola, N., Thierry, M., Sereno, D., Bongiorno, G., Bianchi, R., Khoury, C., Tsirigotakis, N., … Gradoni, L. (2016). Seasonal Dynamics of Phlebotomine Sand Fly Species Proven Vectors of Mediterranean Leishmaniasis Caused by Leishmania infantum. *PLoS Neglected Tropical Diseases*, *10*(2). https://doi.org/10.1371/JOURNAL.PNTD.0004458

Benabid, M., Ghrab, J., Rhim, A., Ben-Romdhane, R., Aoun, K., & Bouratbine, A. (2017). Temporal dynamics and Leishmania infantum infection prevalence of Phlebotomus perniciosus (Diptera, Phlebotominae) in highly endemic areas of visceral leishmaniasis in Tunisia. *PLoS ONE*, *12*(9). https://doi.org/10.1371/journal.pone.0184700

Bennai, K., Tahir, D., Lafri, I., Bendjaballah-Laliam, A., Bitam, I., & Parola, P. (2018). Molecular detection of Leishmania infantum DNA and host blood meal identification in Phlebotomus in a hypoendemic focus of human leishmaniasis in northern Algeria. *PLoS Neglected Tropical Diseases*, *12*(6). https://doi.org/10.1371/journal.pntd.0006513

Cazan, C. D., Păstrav, I. R., Ionică, A. M., Oguz, G., Erisoz Kasap, O., Dvorak, V., Halada, P., Dumitrache, M. O., Volf, P., Alten, B., & Mihalca, A. D. (2019). Updates on the distribution and diversity of sand flies (Diptera: Psychodidae) in Romania. *Parasites and Vectors*, *12*(1). https://doi.org/10.1186/s13071-019-3507-7

Dokhan, M. R., Kenawy, M. A., Doha, S. A., El-Hosary, S. S., Shaibi, T., & Annajar, B. B. (2016). Entomological studies of phlebotomine sand flies (Diptera: Psychodidae) in relation to cutaneous leishmaniasis transmission in Al Rabta, North West of Libya. *Acta Tropica*, *154*, 95–101. https://doi.org/10.1016/J.ACTATROPICA.2015.11.004

Dvorak, V., Kasap, O. E., Ivovic, V., Mikov, O., Stefanovska, J., Martinkovic, F., Omeragic, J., Pajovic, I., Baymak, D., Oguz, G., Hlavackova, K., Gresova, M., Gunay, F., Vaselek, S., Ayhan, N., Lestinova, T., Cvetkovikj, A., Soldo, D. K., Katerinova, I., … Alten, B. (2020). Sand flies (Diptera: Psychodidae) in eight Balkan countries: historical review and region-wide entomological survey. *Parasites and Vectors*, *13*(1), 1–15. https://doi.org/10.1186/S13071-020-04448-W/TABLES/3

Dvořák, V., Tsirigotakis, N., Pavlou, C., Dokianakis, E., Akhoundi, M., Halada, P., Volf, P., Depaquit, J., & Antoniou, M. (2020). Sand fly fauna of Crete and the description of Phlebotomus (Adlerius) creticus n. sp. (Diptera: Psychodidae). *Parasites and Vectors*, *13*(1). https://doi.org/10.1186/s13071-020-04358-x

González, E., Jiménez, M., Hernández, S., Martín-Martín, I., & Molina, R. (2017). Phlebotomine sand fly survey in the focus of leishmaniasis in Madrid, Spain (2012-2014): Seasonal dynamics, Leishmania infantum infection rates and blood meal preferences. *Parasites and Vectors*, *10*(1). https://doi.org/10.1186/s13071-017-2309-z

Gradoni, L., Ferroglio, E., Zanet, S., Mignone, W., Venco, L., Bongiorno, G., Fiorentino, E., Cassini, R., Grillini, M., Simonato, G., Michelutti, A., Montarsi, F., Natale, A., Gizzarelli, M., Foglia Manzillo, V., Solari Basano, F., Nazzari, R., Melideo, O., Gatti, D., & Oliva, G. (2022). Monitoring and detection of new endemic foci of canine leishmaniosis in northern continental

Italy: An update from a study involving five regions (2018–2019). *Veterinary Parasitology: Regional Studies and Reports*, *27*, 100676. https://doi.org/10.1016/J.VPRSR.2021.100676

Kavur, H., Arikan, H., & Ozbel, Y. (2018). Phlebotomus halepensis (Diptera: Psychodidae) vectorial capacity in Afyon and Nigde Province, Turkey. *Journal of Medical Entomology*, *55*(2), 317–322. https://doi.org/10.1093/jme/tjx210

Kavur, H., Artun, O., Evyapan, G., Demirkazık, M., Alptekin, D., & Koltaş, İ. S. (2018). Sand fly fauna and environmental parameters in a cutaneous leishmaniasis endemic region in Karaisali, Adana, Turkey. *Cukurova Medical Journal*, *43*(4), 1–1. https://doi.org/10.17826/cumj.336142

Kniha, E., Dvořák, V., Halada, P., Milchram, M., Obwaller, A. G., Kuhls, K., Schlegel, S., Köhsler, M., Poeppl, W., Bakran-Lebl, K., Fuehrer, H. P., Volfová, V., Mooseder, G., Ivovic, V., Volf, P., & Walochnik, J. (2020). Integrative Approach to Phlebotomus mascittii Grassi, 1908: First Record in Vienna with New Morphological and Molecular Insights. *Pathogens 2020, Vol. 9, Page 1032*, *9*(12), 1032. https://doi.org/10.3390/PATHOGENS9121032

Kniha, E., Milchram, M., Dvořák, V., Halada, P., Obwaller, A. G., Poeppl, W., Mooseder, G., Volf, P., & Walochnik, J. (2021). Ecology, seasonality and host preferences of Austrian Phlebotomus (Transphlebotomus) mascittii Grassi, 1908, populations. *Parasites and Vectors*, *14*(1), 1–12. https://doi.org/10.1186/S13071-021-04787-2/TABLES/4

Kuhls, K., Moskalenko, O., Sukiasyan, A., Manukyan, D., Melik-Andreasyan, G., Atshemyan, L., Apresyan, H., Strelkova, M., Jaeschke, A., Wieland, R., Frohme, M., Cortes, S., & Keshishyan, A. (2021). Microsatellite based molecular epidemiology of leishmania infantum from re-emerging foci of visceral leishmaniasis in armenia and pilot risk assessment by ecological niche modeling. *PLoS Neglected Tropical Diseases*, *15*(4). https://doi.org/10.1371/journal.pntd.0009288

Morosetti, G., Toson, M., Trevisiol, K., Idrizi, I., Natale, A., Lucchese, L., Michelutti, A., Ceschi, P., Lorenzi, G., Piffer, C., Fiorentino, E., Bongiorno, G., & Gradoni, L. (2020). Canine leishmaniosis in the Italian northeastern Alps: A survey to assess serological prevalence in dogs and distribution of phlebotomine sand flies in the Autonomous Province of Bolzano - South Tyrol, Italy. *Veterinary Parasitology: Regional Studies and Reports*, *21*, 100432. https://doi.org/10.1016/J.VPRSR.2020.100432

Muñoz, C., Martínez-de la Puente, J., Figuerola, J., Pérez-Cutillas, P., Navarro, R., Ortuño, M., Bernal, L. J., Ortiz, J., Soriguer, R., & Berriatua, E. (2019). Molecular xenomonitoring and host identification of Leishmania sand fly vectors in a Mediterranean periurban wildlife park. *Transboundary and Emerging Diseases*, *66*(6), 2546–2561. https://doi.org/10.1111/tbed.13319

Muñoz, C., Risueño, J., Pérez-Cutillas, P., Bernal, L. J., Ortiz, J. M., Ruiz de Ybáñez, R., Sánchez-López, P. F., Martínez-Carrasco, C., Del Río, L., De la Rúa, P., García-Martínez, J. D., Gonzálvez, M., Murcia, L., Collantes, F., Goyena, E., Spitzova, T., Elshanat, S., & Berriatua, E. (2021). Density assessment and reporting for Phlebotomus perniciosus and other sand fly species in periurban residential estates in Spain. *Parasitology Research*, *120*(9), 3091–3103. https://doi.org/10.1007/s00436-021-07270-0

MuÑoz, C., RisueÑo, J., Yilmaz, A., PÉrez-Cutillas, P., Goyena, E., OrtuÑo, M., Bernal, L. J., Ortiz, J., Alten, B., & Berriatua, E. (2018). Investigations of Phlebotomus perniciosus sand flies in rural Spain reveal strongly aggregated and gender-specific spatial distributions and advocate use of light-attraction traps. *Medical and Veterinary Entomology*, *32*(2), 186–196. https://doi.org/10.1111/mve.12275

Orshan, L., Elbaz, S., Ben-Ari, Y., Akad, F., Afik, O., Ben-Avi, I., Dias, D., Ish-Shalom, D., Studentsky, L., & Zonstein, I. (2016). Distribution and Dispersal of Phlebotomus papatasi (Diptera: Psychodidae) in a Zoonotic Cutaneous Leishmaniasis Focus, the Northern Negev, Israel. *PLoS Neglected Tropical Diseases*, *10*(7). https://doi.org/10.1371/journal.pntd.0004819

Pekağırbaş, M., Karakuş, M., Kasap, O. E., Demir, S., Nalçacı, M., Töz, S., Eren, H., & Özbel, Y. (2021). Investigation of Phlebotominae (Diptera: Psychodidae) Fauna, Seasonal Dynamics, and Natural Leishmania spp. Infection in Muğla, Southwest of Turkey. *Acta Tropica*, *216*. https://doi.org/10.1016/j.actatropica.2021.105827

Prudhomme, J. (2015). *Phlébotomes et écosystèmes : impact des facteurs biotiques et abiotiques sur la structure génétique et phénotypique des populations*. https://theses.hal.science/tel-01414996

Prudhomme, J., Rahola, N., Toty, C., Cassan, C., Roiz, D., Vergnes, B., Thierry, M., Rioux, J. A., Alten, B., Sereno, D., & Bañuls, A. L. (2015). Ecology and spatiotemporal dynamics of sandflies in the Mediterranean Languedoc region (Roquedur area, Gard, France). *Parasites and Vectors*, *8*(1), 1–14. https://doi.org/10.1186/S13071-015-1250-2/TABLES/6

Şuleşco, T., Erisoz Kasap, O., Halada, P., Oğuz, G., Rusnac, D., Gresova, M., Alten, B., Volf, P., & Dvorak, V. (2021). Phlebotomine sand fly survey in the Republic of Moldova: species composition, distribution and host preferences. *Parasites and Vectors*, *14*(1). https://doi.org/10.1186/s13071-021-04858-4

Tsirigotakis, N., Pavlou, C., Christodoulou, V., Dokianakis, E., Kourouniotis, C., Alten, B., & Antoniou, M. (2018). Phlebotomine sand flies (Diptera: Psychodidae) in the Greek Aegean Islands: Ecological approaches. *Parasites and Vectors*, *11*(1). https://doi.org/10.1186/s13071-018-2680-4

Vaselek, S., Ayhan, N., Oguz, G., Erisoz Kasap, O., Savić, S., Di Muccio, T., Gradoni, L., Ozbel, Y., Alten, B., & Petrić, D. (2017). Sand fly and Leishmania spp. survey in Vojvodina (Serbia): First detection of Leishmania infantum DNA in sand flies and the first record of Phlebotomus (Transphlebotomus) mascittii Grassi, 1908. *Parasites and Vectors*, *10*(1). https://doi.org/10.1186/s13071-017-2386-z

Vaselek, S., Dvorak, V., Hlavackova, K., Ayhan, N., Halada, P., Oguz, G., Ivović, V., Ozbel, Y., Charrel, R. N., Alten, B., & Petrić, D. (2019). A survey of sand flies (Diptera, Phlebotominae) along recurrent transit routes in Serbia. *Acta Tropica*, *197*. https://doi.org/10.1016/j.actatropica.2019.105063
