# Supplementary material for: Understanding and predicting the geographic distributions of phlebotomine sand flies in and around Europe
Source: Clim Change. 2025 Nov 5;178(11):205. doi: 10.1007/s10584-025-04009-z (PMC12589297; doi:10.1007/s10584-025-04009-z)
Supplement: Supplementary file 4 — Supplementary file4 (PDF 91 KB) [file 10584_2025_4009_MOESM4_ESM.pdf]

Supplementary Information 4. Regularization multiplier and spatial cross-validation

We determined the values of regularization multiplier per species by fitting single-variable models with regularization multiplier one, three, five, seven and nine, and then inspecting the smoothness of response curves(Elith et al., 2011; Radosavljevic & Anderson, 2014). Abrupt / multimodal response curves for many variables were considered to be an indicator of overfitting(Zhu & Qiao, 2016); regularization multiplier values that produced smooth response curves were chosen.

We divided all points into five spatial clusters using the function cv_cluster() with the 'blockCV' package(Valavi et al., 2019) and held out each cluster once for model evaluation. Only two species (i.e., *Ph. papatasi* and *Ph. perfiliewi*) were observed in widespread areas (after environmental thinning) that allowed for cross-validation with five spatial blocks. Three species had four folds cross-validation, five species were cross-validated with three spatial blocks, and two species (i.e., *Ph. tobbi, Ph. ariasi*) could only be cross-validated with two folds due to paucity of species occurrence records.

Table. The chosen regularization multiplier for model fitting and the folds of spatial cross-validation per Phlebotomus species.

Species Name	Regularization multiplier	Folds
Ph. papatasi	9	5
Ph. perniciosus	9	3
S. minuta	9	4
Ph. sergenti	9	4
Ph. tobbi	9	2
Ph. mascittii	9	3
Ph. neglectus	7	3
Ph. perfiliewi	7	5
Ph. ariasi	9	2
S. dentata	9	3
Ph. simici	7	3
Ph. alexandri	5	4

Reference

Elith, J., Phillips, S. J., Hastie, T., Dudík, M., Chee, Y. E., & Yates, C. J. (2011). A statistical explanation of MaxEnt for ecologists. *Diversity and Distributions*, *17*(1), 43–57. https://doi.org/10.1111/j.1472-4642.2010.00725.x

Radosavljevic, A., & Anderson, R. P. (2014). Making better Maxent models of species distributions: Complexity, overfitting and evaluation. *Journal of Biogeography*, *41*(4), 629–643. https://doi.org/10.1111/jbi.12227

Valavi, R., Elith, J., Lahoz-Monfort, J. J., & Guillera-Arroita, G. (2019). blockCV: An r package for generating spatially or environmentally separated folds for k-fold cross-validation of species distribution models. *Methods in Ecology and Evolution*, *10*(2), 225–232. https://doi.org/10.1111/2041-210X.13107

Zhu, G., & Qiao, H. (2016). Effect of the Maxent model’s complexity on the prediction of species potential distributions. *Biodiversity Science*, *24*(10), 1189–1196. https://doi.org/10.17520/biods.2016265
