# Supplementary material for: Understanding and predicting the geographic distributions of phlebotomine sand flies in and around Europe
Source: Clim Change. 2025 Nov 5;178(11):205. doi: 10.1007/s10584-025-04009-z (PMC12589297; doi:10.1007/s10584-025-04009-z)
Supplement: Supplementary file 5 — Supplementary file5 (PDF 124 KB) [file 10584_2025_4009_MOESM5_ESM.pdf]

Supplementary Information 5. Method for Effect Size Computation

We computed the effect size of variables using the lambdas file of Maxent model[1] together with the training dataset. First, the coefficient estimates (i.e., the lambdas) and the feature class were extracted from the lambdas file using parse_lambdas() function of package 'rmaxent'[2]. This subset of the lambdas file was then combined with all presence observations from the training dataset, so that variable values were paired up with lambdas per variable per feature. Assuming x = predictor variables, an intermediate value of effect size was calculated for each row, that is λ for each raw feature and λ * 2 * x for each quadradic feature. For product features, it is λ * 2 * the sign of the product * square root of the absolute value of the product. For forward hinge feature, if x < hinge then 0 otherwise λ. For backward hinge feature, if x < hinge then λ. An intermediate value of a threshold feature can also be calculated similarly, if x < threshold then 0 otherwise λ, although we excluded threshold features from our study[3]. Afterwards, the intermediate values were summed per sampling point so that the effects of features were netted at each observation record. Finally, the effect size was the mean of the absolute netted values across all points. In our study, we computed effect size per model instead of per variable.

To demonstrate in R[4]:

```
library(tidyverse)

x1 <- 1:5                    # predictor variable 1
x2 <- c(-1.5, -1, 0, 1, 1.5)   # predictor variable 2

y1 = -0.5*x1^2                              # quadradic feature
y2 = case_when(x1<2.5 ~ 0, x1>=2.5 ~ x1)  # hinge feature
y3 = 2*x1*x2                           # product feature
y4 = y1+y2+y3                             # all features

l1 = -0.5*2*x1                          # slope of the quadradic feature
l2 = case_when(x1<2.5 ~ 0, x1>=2.5 ~ 1)  # slope of the linear feature
l3 = 2*2*sign(x1*x2)*sqrt(abs(x1*x2))  # slope of the product feature
l=l1+l2+l3                             # intermediate value
size = sum(abs(l))/5 # effect size is the mean of absolute values across points

par(mfrow=c(2,2)) # plot
plot (x1, y1, ylab = "y1, quadradic feature")
plot (x1, y2, ylab = "y2, forward hinge feature")
plot (sign(x1*x2)*sqrt(abs(x1*x2)), y3, ylab = "y3, product feature")
plot(x1, y4, ylab = "y4, all features")
lines(1:5, size*(1:5)-15, col = "blue", lwd = 2)  # A line to show size
text(3, 6, labels = paste0("slope shows the effect size = ",round(size,2)),
    col = "blue", cex = 1)
```

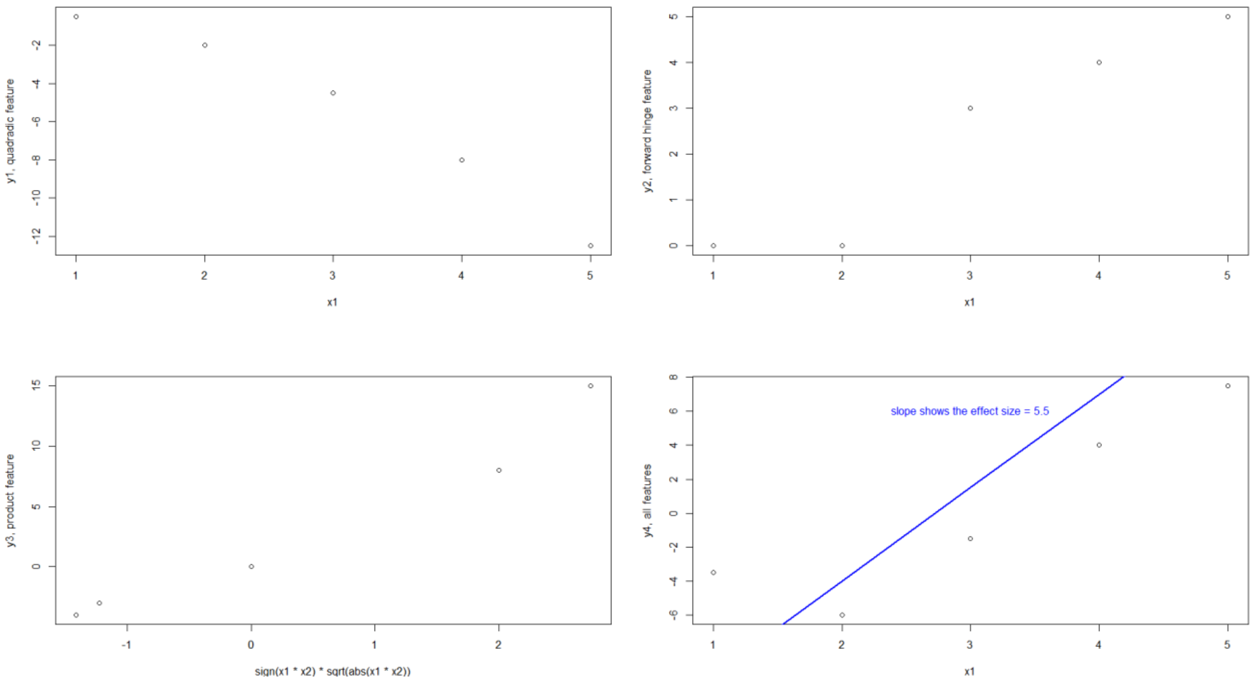

Reference

1. Phillips, S. J. A Brief Tutorial on Maxent. http://biodiversityinformatics.amnh.org/open_source/maxent/. (2017).

2. Baumgartner J & Wilson P. _rmaxent: Tools for working with Maxent in R_. R package version 0.8.5.9000. Preprint at https://github.com/johnbaums/rmaxent.

3. Phillips, S. J., Anderson, R. P., Dudík, M., Schapire, R. E. & Blair, M. E. Opening the black box: an open-source release of Maxent. *Ecography* 40, 887–893 (2017).

4. R Core Team. R: A language and environment for statistical computing. Preprint at https://www.R-project.org/ (2023).
