# Supplementary figures and images for: Understanding and predicting the geographic distributions of phlebotomine sand flies in and around Europe
Source: Clim Change. 2025 Nov 5;178(11):205. doi: 10.1007/s10584-025-04009-z (PMC12589297; doi:10.1007/s10584-025-04009-z)

cmi\_09\_std

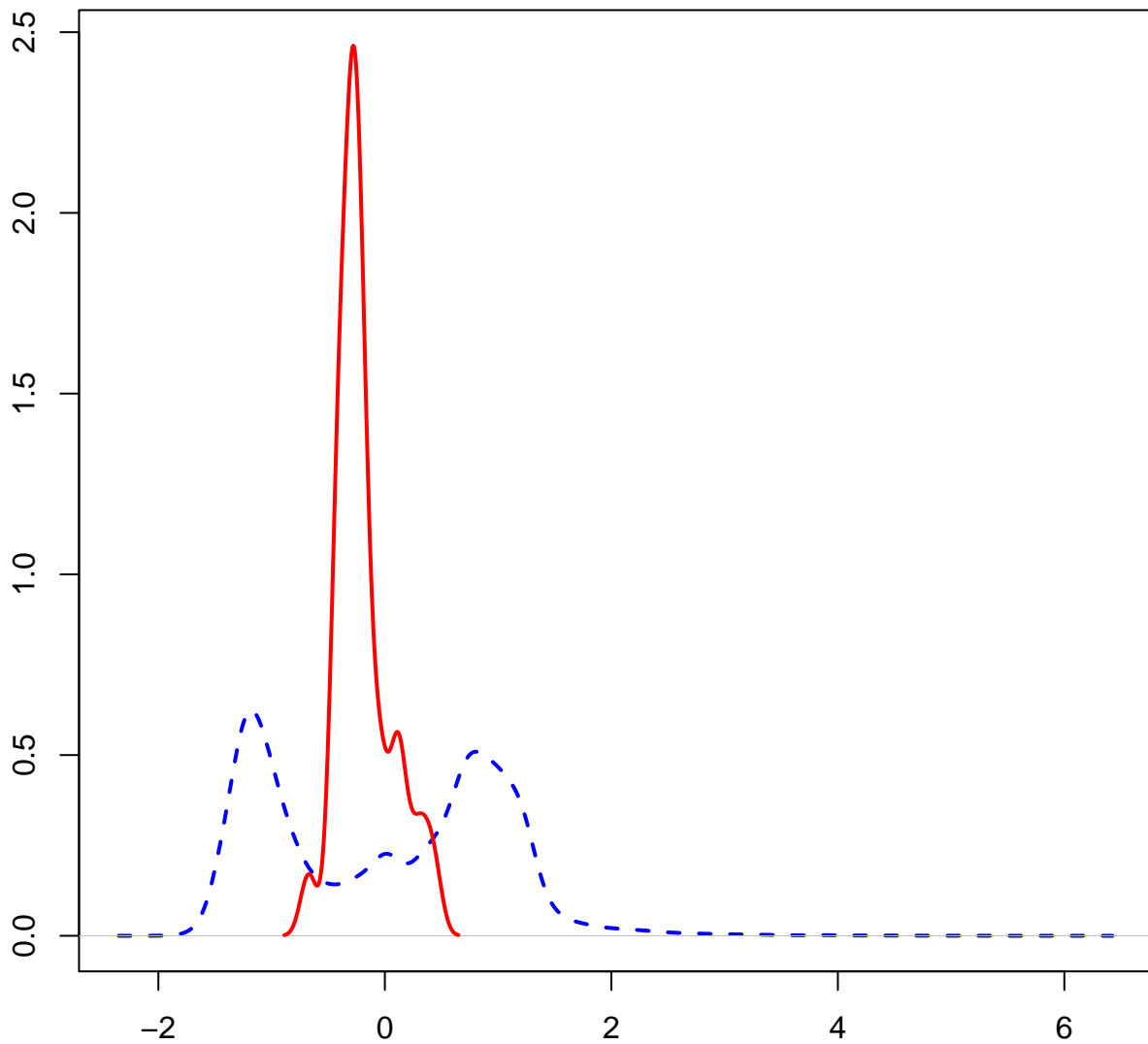

Supplement: Supplementary file 6 — Supplementary file6 (ZIP 115 KB) [file 10584_2025_4009_MOESM6_ESM.zip › SI6/1_Ph.alexandri_cmi_09_std.pdf]

bio1\_std

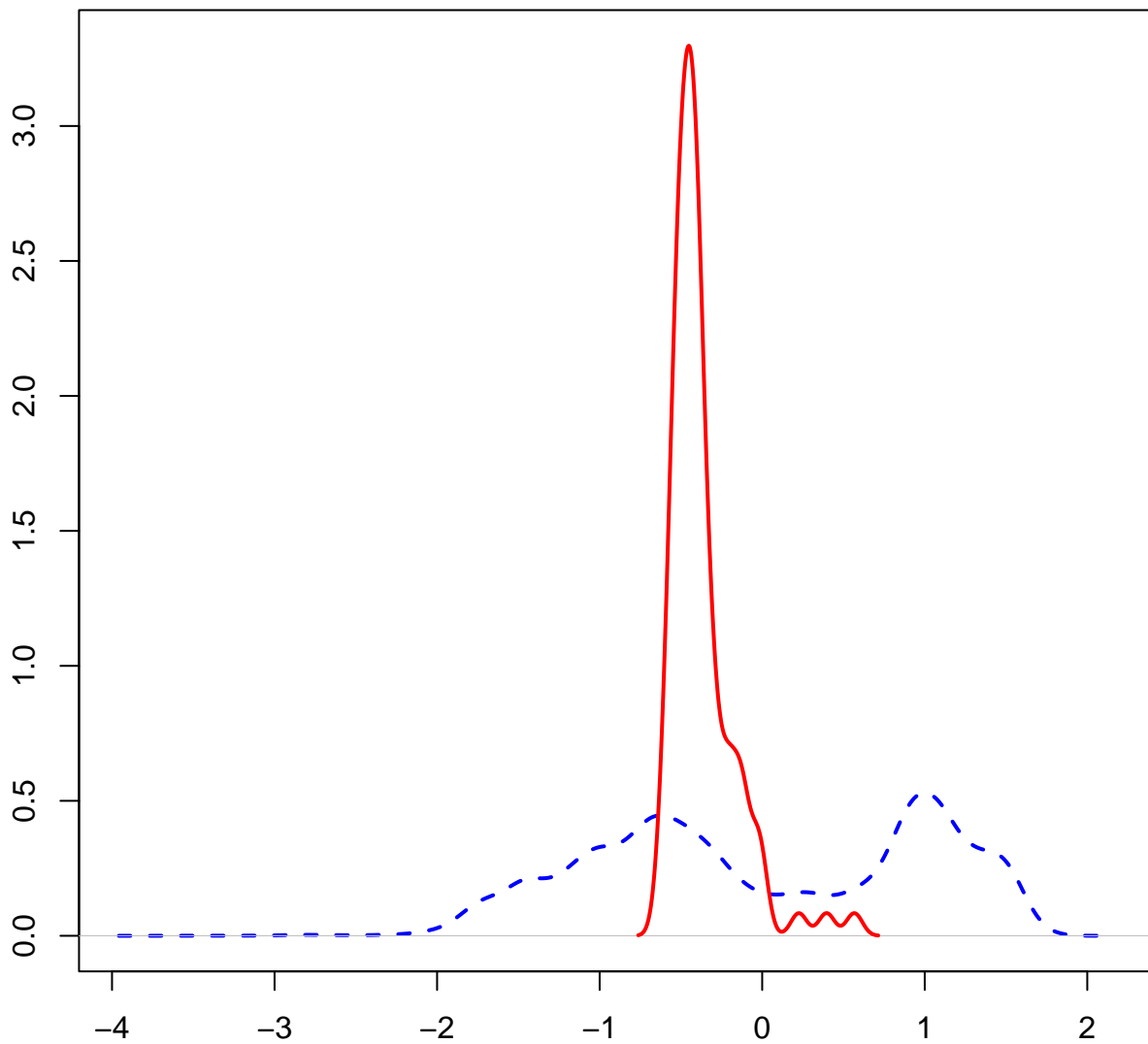

Supplement: Supplementary file 6 — Supplementary file6 (ZIP 115 KB) [file 10584_2025_4009_MOESM6_ESM.zip › SI6/13_Ph.mascittii_bio1_std.pdf]

cmi\_08\_std

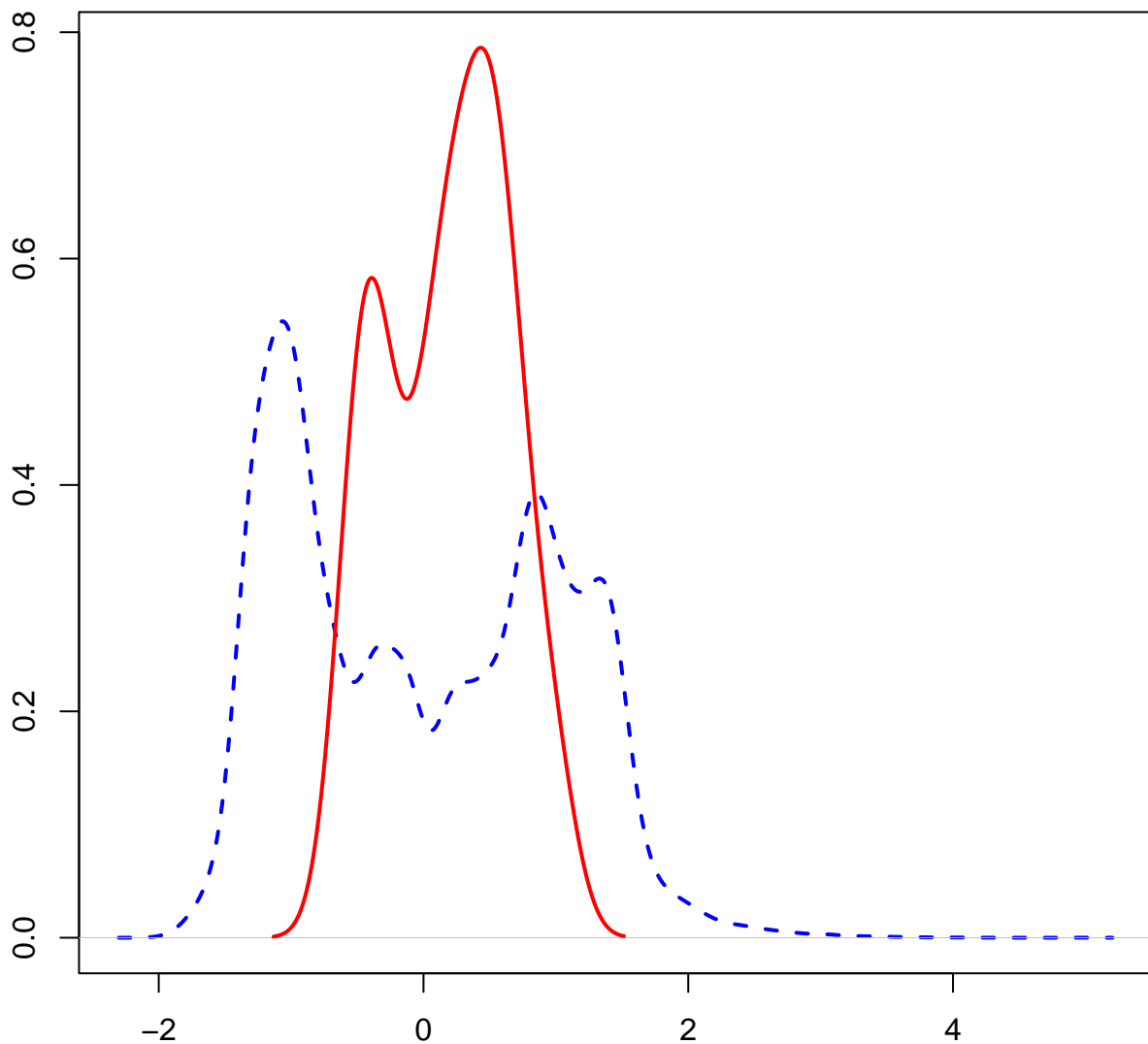

Supplement: Supplementary file 6 — Supplementary file6 (ZIP 115 KB) [file 10584_2025_4009_MOESM6_ESM.zip › SI6/16_Ph.neglectus_cmi_08_std.pdf]

pet\_penman\_04\_std

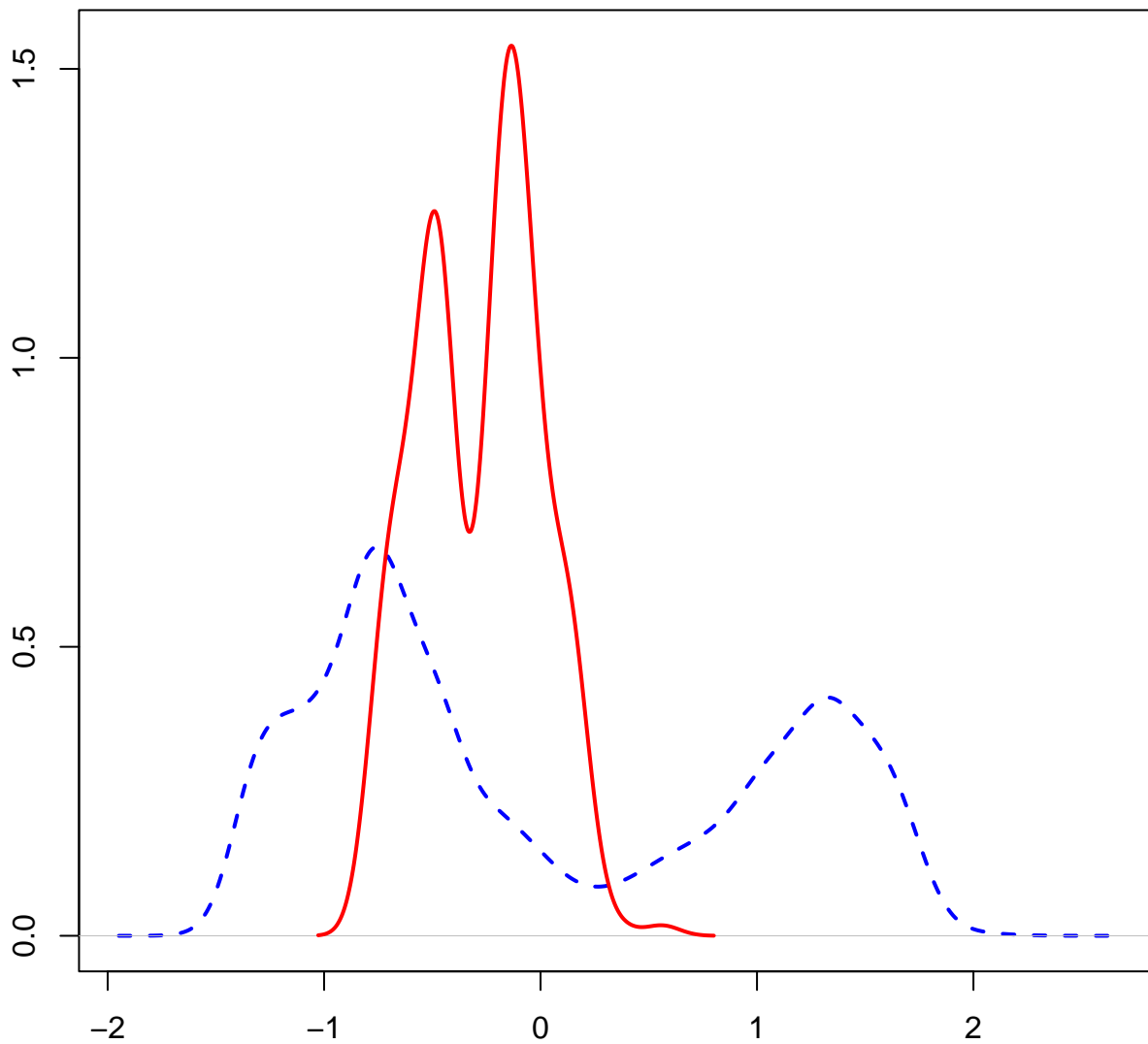

Supplement: Supplementary file 6 — Supplementary file6 (ZIP 115 KB) [file 10584_2025_4009_MOESM6_ESM.zip › SI6/19_Ph.papatasi_pet_penman_04_std.pdf]

cmi\_06\_std

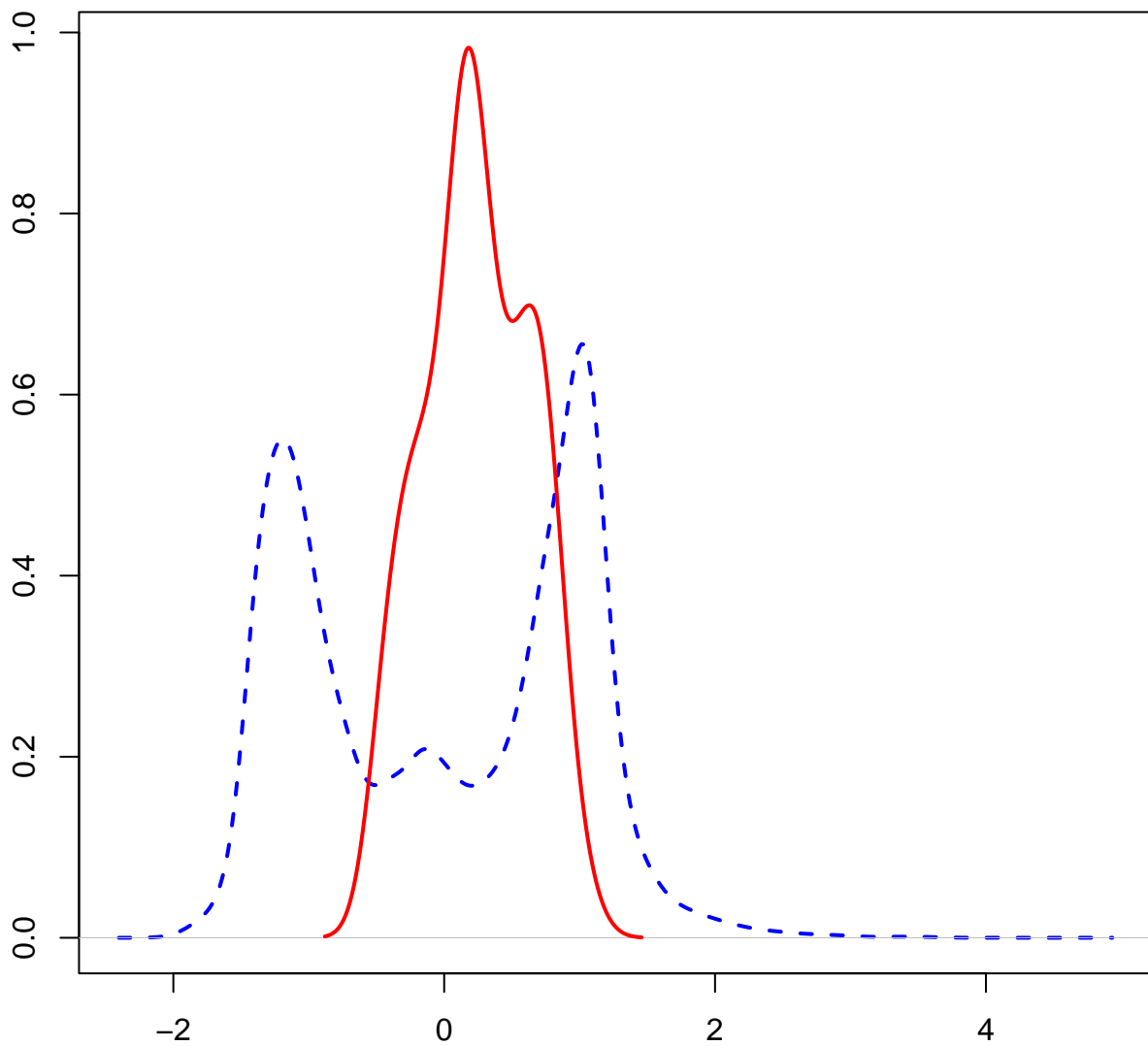

Supplement: Supplementary file 6 — Supplementary file6 (ZIP 115 KB) [file 10584_2025_4009_MOESM6_ESM.zip › SI6/22_Ph.perfiliewi_cmi_06_std.pdf]

tas\_12\_std

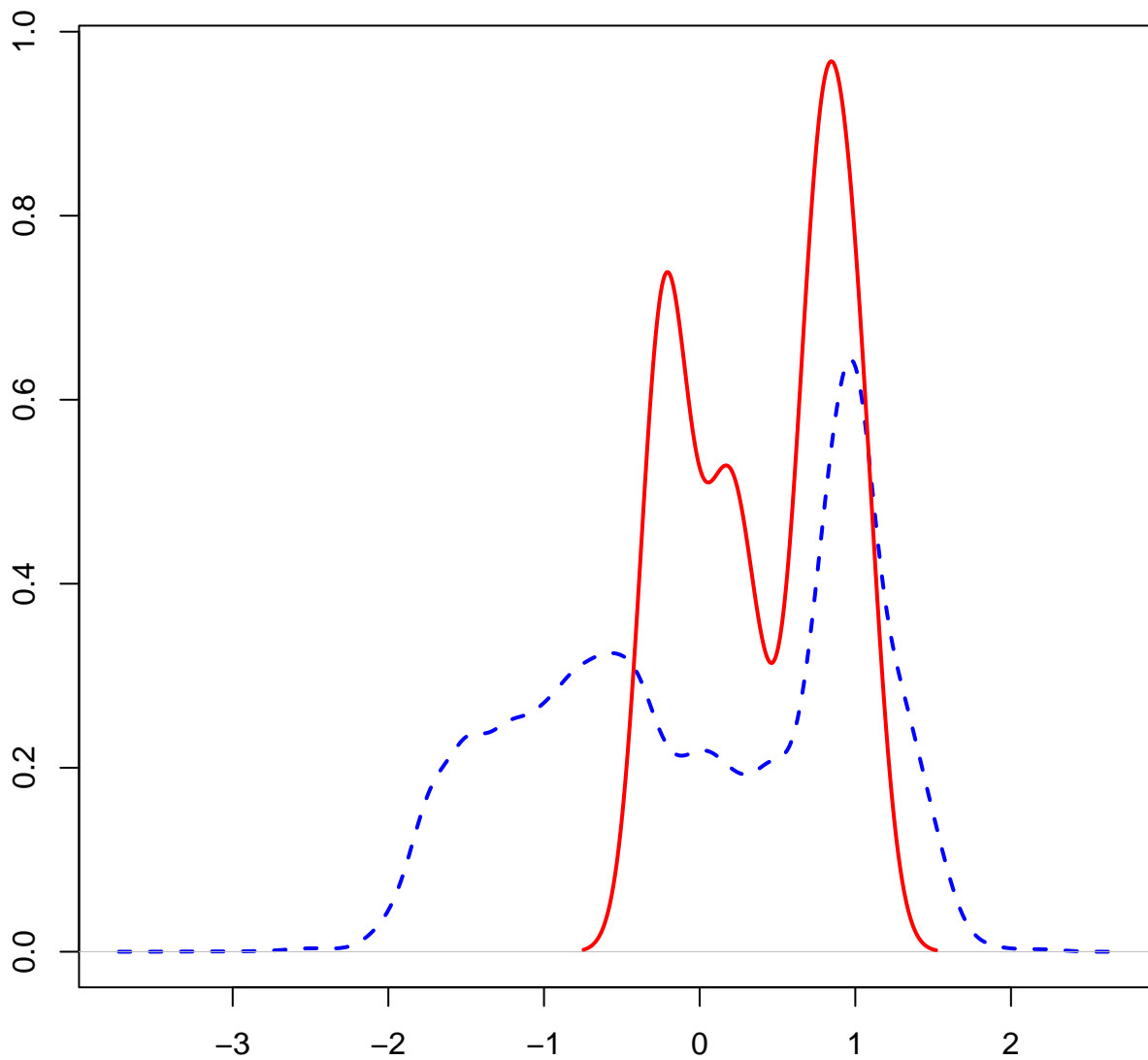

Supplement: Supplementary file 6 — Supplementary file6 (ZIP 115 KB) [file 10584_2025_4009_MOESM6_ESM.zip › SI6/25_Ph.perniciosus_tas_12_std.pdf]

vpd\_min\_std

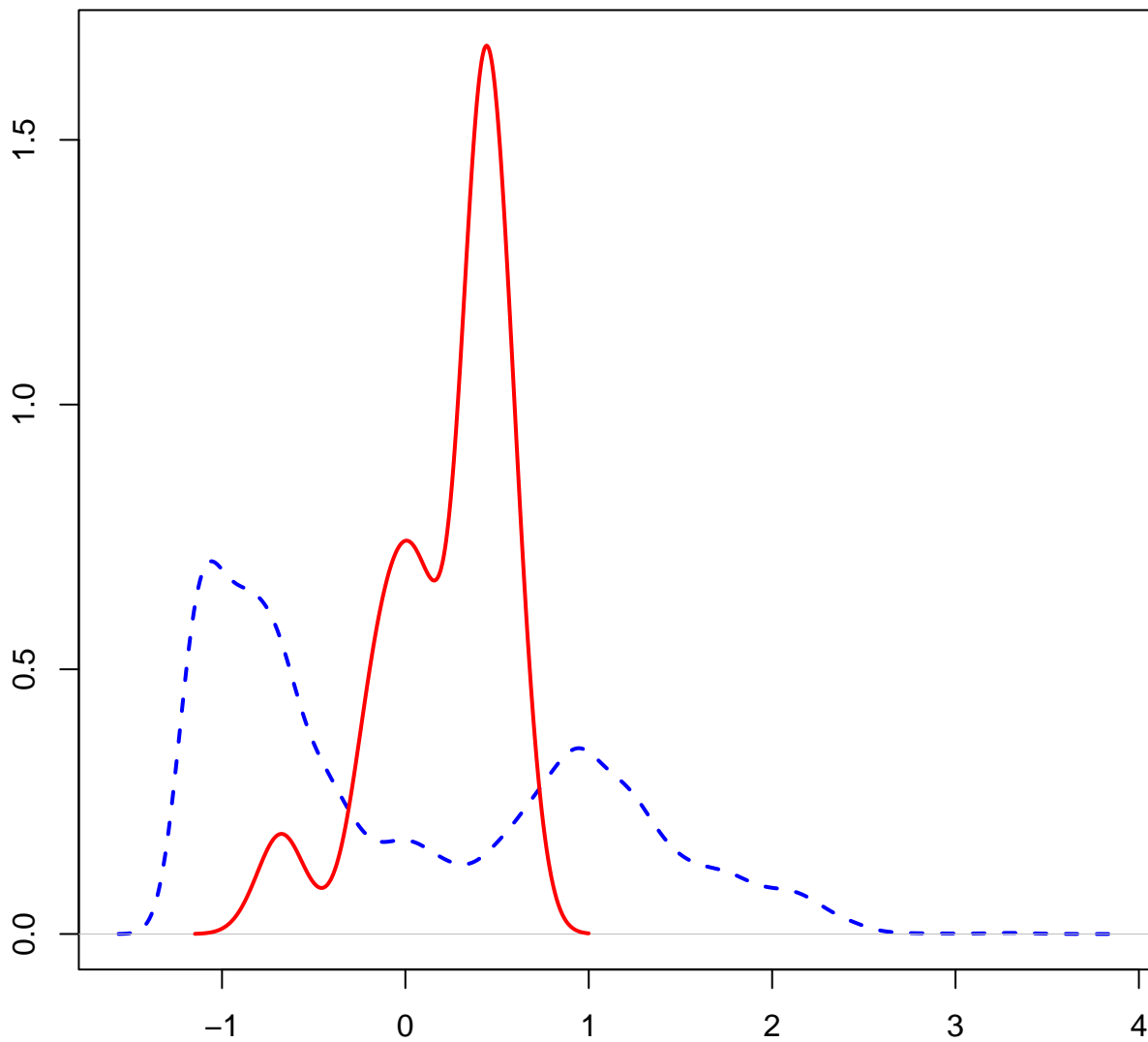

Supplement: Supplementary file 6 — Supplementary file6 (ZIP 115 KB) [file 10584_2025_4009_MOESM6_ESM.zip › SI6/28_Ph.sergenti_vpd_min_std.pdf]

cmi\_09\_std

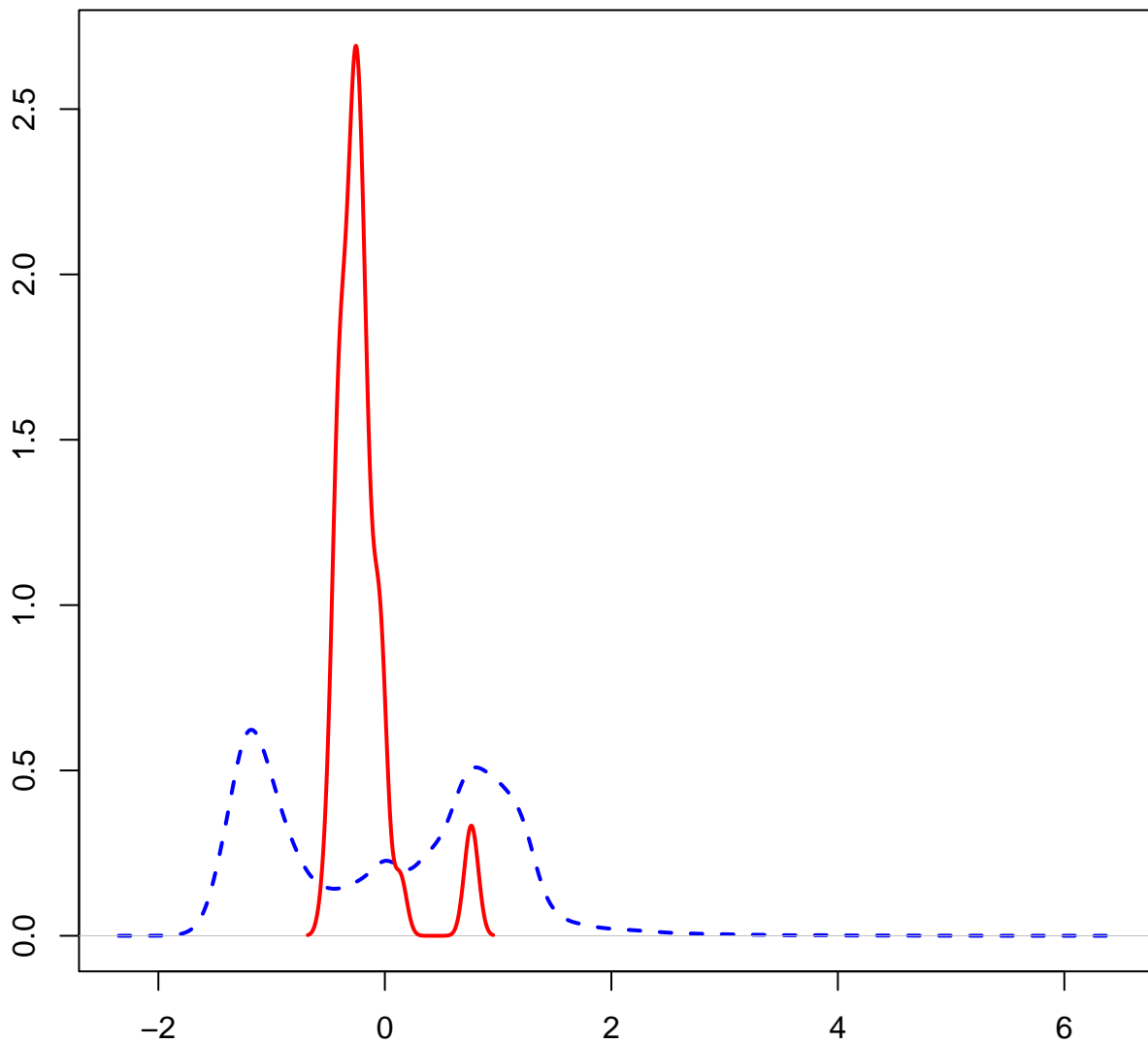

Supplement: Supplementary file 6 — Supplementary file6 (ZIP 115 KB) [file 10584_2025_4009_MOESM6_ESM.zip › SI6/31_Ph.simici_cmi_09_std.pdf]

cmi\_range\_std

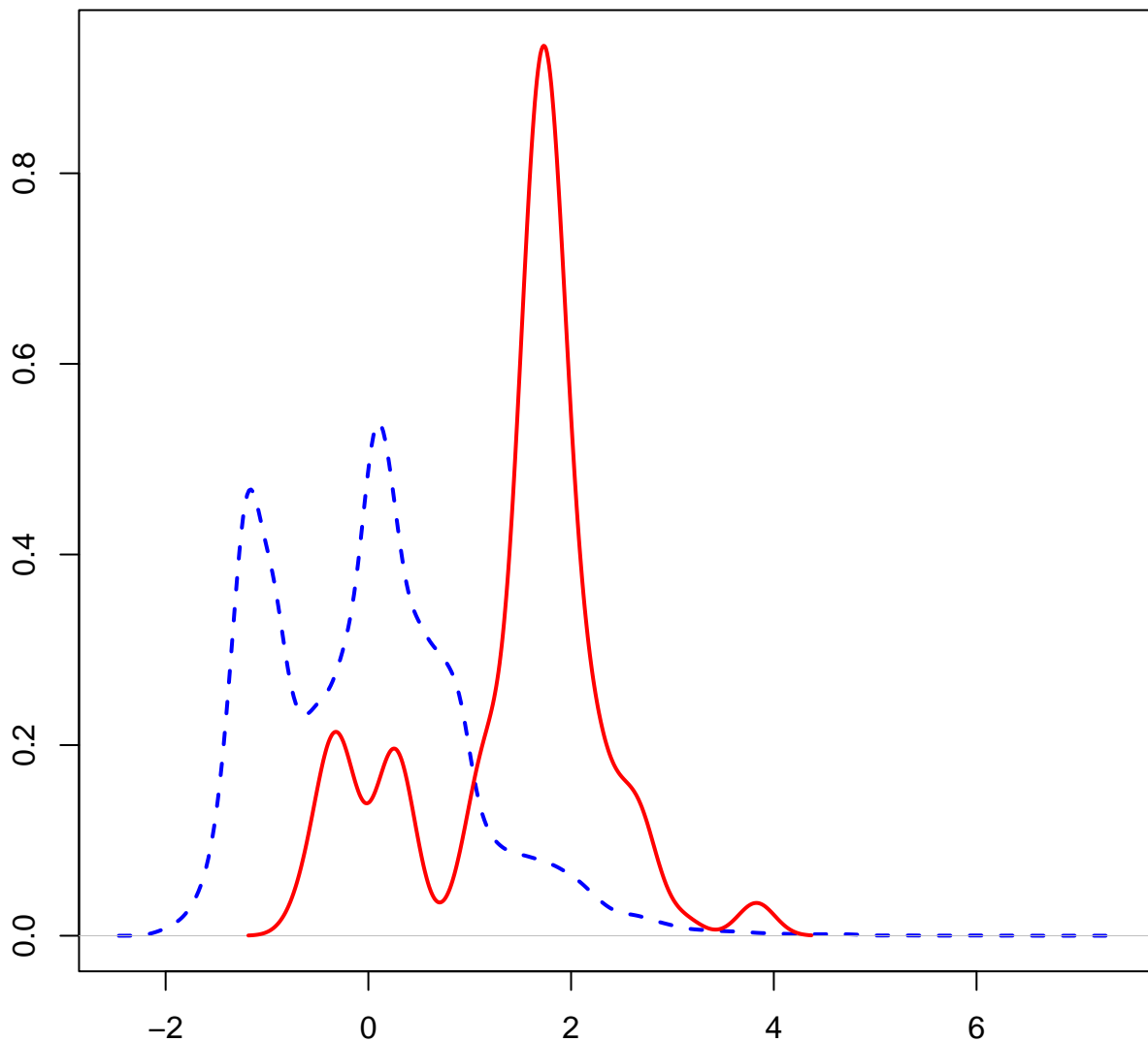

Supplement: Supplementary file 6 — Supplementary file6 (ZIP 115 KB) [file 10584_2025_4009_MOESM6_ESM.zip › SI6/37_Ph.tobbi_cmi_range_std.pdf]

cmi\_06\_std

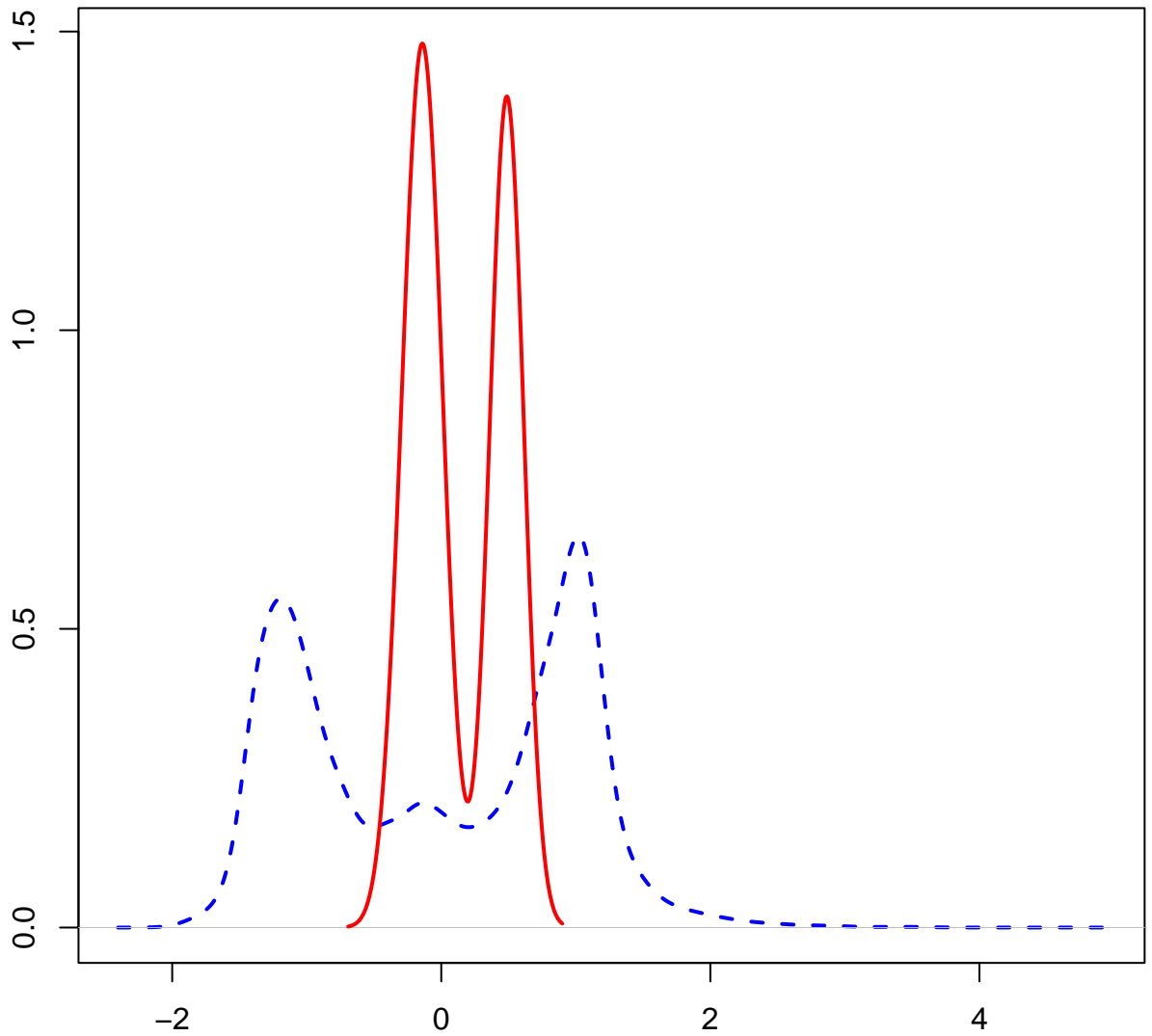

Supplement: Supplementary file 6 — Supplementary file6 (ZIP 115 KB) [file 10584_2025_4009_MOESM6_ESM.zip › SI6/4_Ph.ariasi_cmi_06_std.pdf]

tas\_10\_std

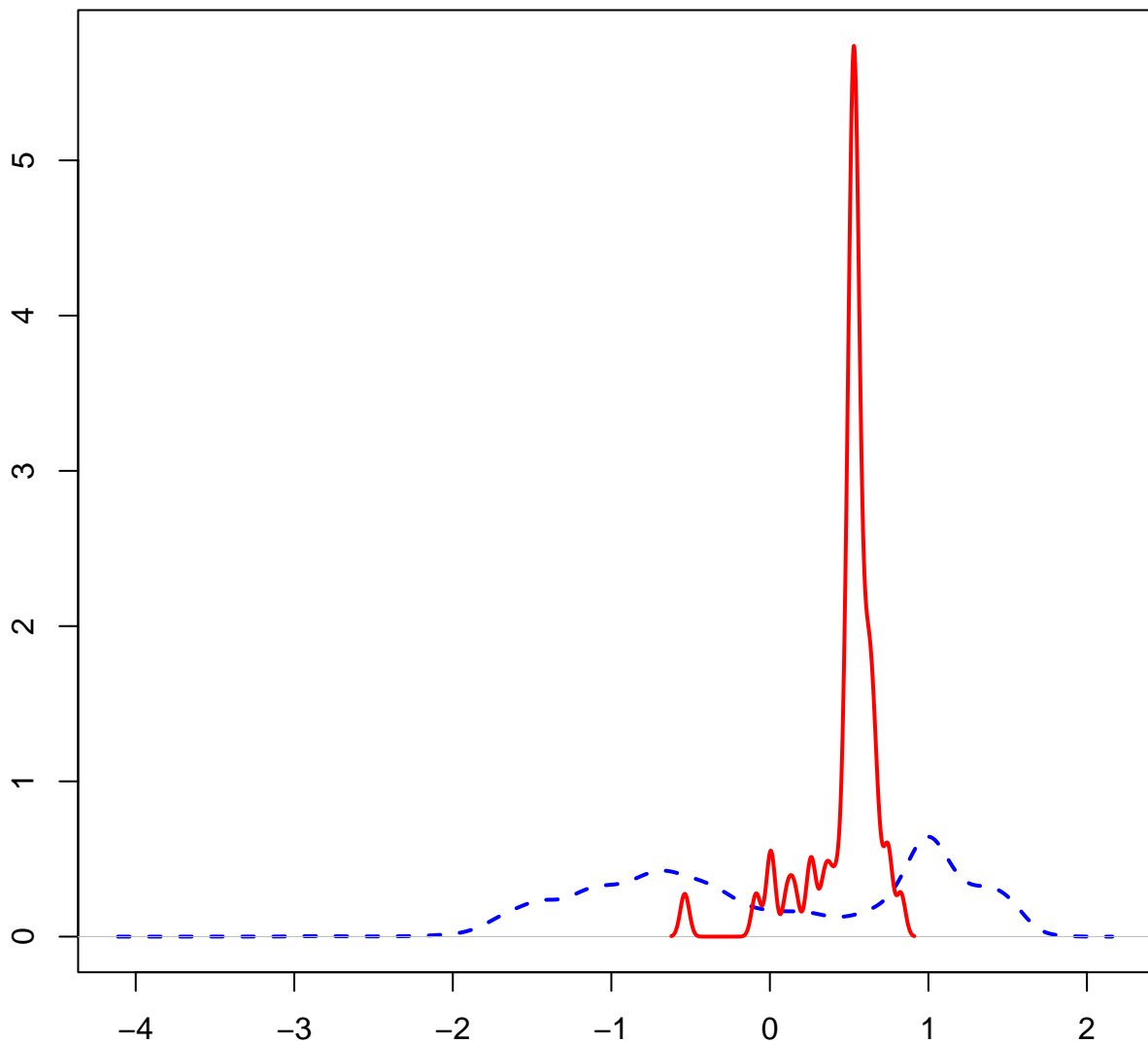

Supplement: Supplementary file 6 — Supplementary file6 (ZIP 115 KB) [file 10584_2025_4009_MOESM6_ESM.zip › SI6/40_S.dentata_tas_10_std.pdf]

cmi\_03\_std

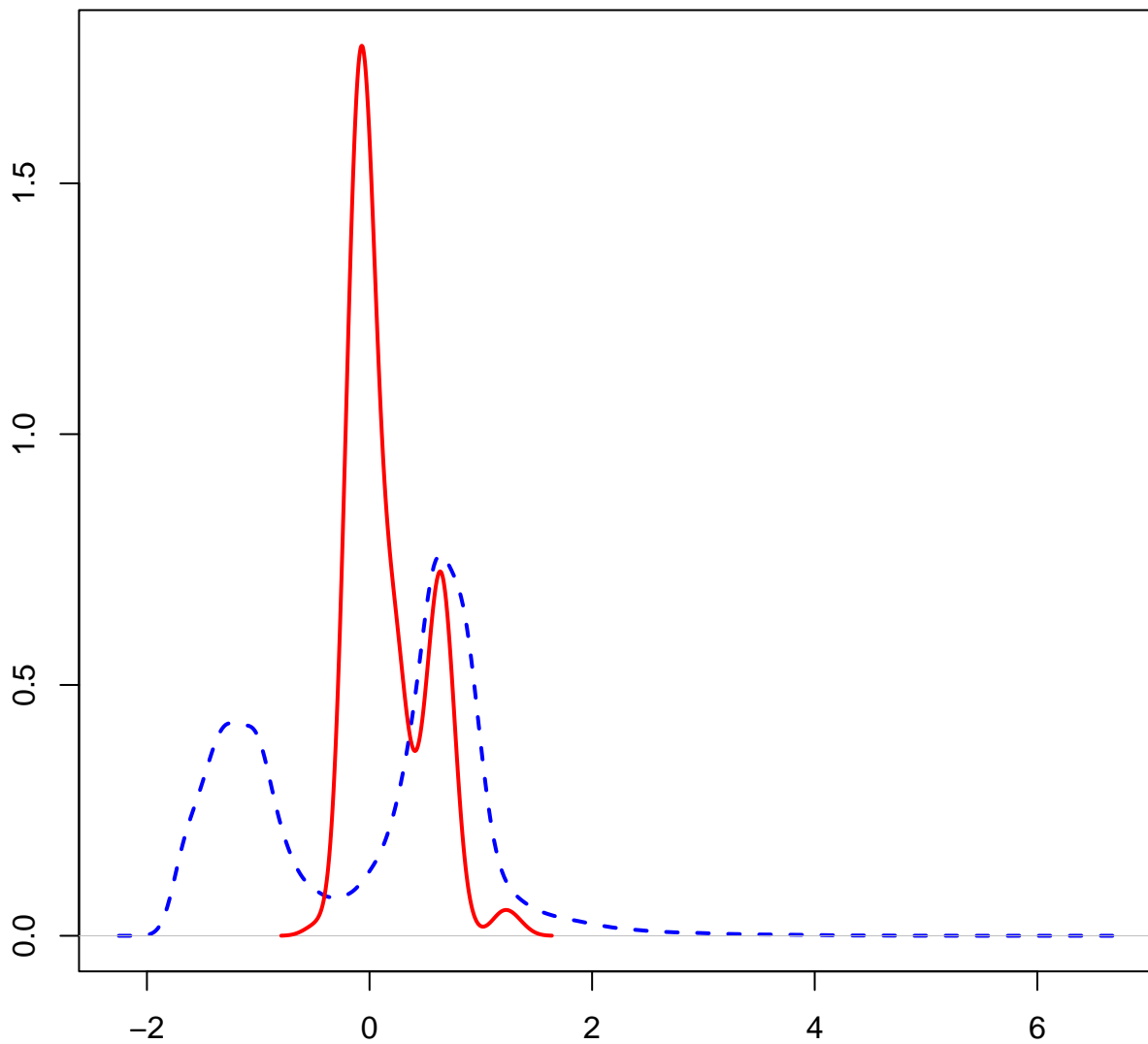

Supplement: Supplementary file 6 — Supplementary file6 (ZIP 115 KB) [file 10584_2025_4009_MOESM6_ESM.zip › SI6/43_S.minuta_cmi_03_std.pdf]
